# Genomic Insights of Emerging Multidrug-Resistant OXA-48-Producing ST135 *Proteus mirabilis*

**DOI:** 10.3390/antibiotics14080750

**Published:** 2025-07-25

**Authors:** Angeliki Mavroidi, Elisavet Froukala, Nick Spanakis, Aikaterini Michelaki, Maria Orfanidou, Vasiliki Koumaki, Athanasios Tsakris

**Affiliations:** 1 Department of Microbiology, General University Hospital of Patras, 26504 Rio, Greece; amavroidi@live.com; 2 Department of Microbiology, Medical School, University of Athens, 11527 Athens, Greece; elisavetfrou@gmail.com (E.F.); nespanakis@gmail.com (N.S.); vkoumaki@med.uoa.gr (V.K.); 3 Department of Microbiology, General Hospital G. Gennimatas, 11527 Athens, Greece; kmichelaki@yahoo.gr (A.M.); m.orfanidou@gna-gennimatas.gr (M.O.)

**Keywords:** *Proteus mirabilis*, carbapenemases, OXA-48, ESBL, MLST, WGS, phylogenomics, comparative genomics

## Abstract

**Background/Objectives**: Among *Enterobacterales*, OXA-48-like-producing *Proteus mirabilis* strains have been scarcely detected. Herein, we characterized a *bla*_OXA-48_-harbouring *P. mirabilis* strain recovered from Greece (Pm GR-1), while phylogenomics and comparative genomics analyses with previously published *bla*_OXA-48_ carriers were also assessed. **Methods**: Characterization of Pm GR-1 was performed by the Vitek^®^ Compact and Mass Spectrometry systems, antimicrobial susceptibility testing, detection of beta-lactamases, multilocus-sequence typing (MLST), and whole-genome sequencing (WGS). In silico prediction of mobile genetic elements (MGEs), genomic islands (GIs), antimicrobial resistance genes (ARGs) and virulence factors (VFs), and phylogenetic, core-genome SNP and comparative genomics analyses were executed using bioinformatic tools. **Results**: Pm GR-1 was isolated from a urine sample of an outpatient in a Greek hospital. It exhibited a multidrug-resistant phenotype, being susceptible only to amikacin and ceftazidime/avibactam. It co-carried several beta-lactamase genes on the chromosome (*bla*_OXA-48_, *bla*_CTX-M-14_, *bla*_TEM-1_) and a plasmid *(bla*_TEM-2_) and several other ARGs, but also mutations associated with quinolone resistance in the DNA gyrase and topoisomerase IV subunits. It belonged to the international clone ST135 that has also been detected among OXA-48-producing *P. mirabilis* strains from Germany and the USA. Pm GR-1 was genetically related to those from Germany, sharing highly similar MGEs, GIs, ARGs and VFs, including the chromosomal *bla*_OXA-48_ genetic structure, the O-antigen locus, the flagella locus, the MR/P fimbriae operon, and the urease gene cluster. **Conclusions**: To our knowledge, this is the first report from Greece of a *bla*_OXA-48_-possessing *P. mirabilis* strain. The emergence of *bla*_OXA-48_ among *P. mirabilis* strains of the international clone ST135 in different geographical regions is worrying. Close monitoring of these strains is required in One Health settings.

## 1. Introduction

*Proteus mirabilis* is commonly found in the intestinal microbiota of humans and animals, as well as in the environment through faecal contamination [1]. It may also be an opportunistic pathogen in humans, causing more frequent urinary tract infections and catheter-associated urinary tract infections, but also bloodstream, wound, and ear infections; rheumatoid arthritis; and meningitis in infants [2]. Infections caused by *P. mirabilis* are often difficult to treat in clinical practice due to its intrinsic and acquired resistance mechanisms to several last-resort antibiotics [3]. In more detail, *P. mirabilis* strains are intrinsically resistant to tetracyclines, colistin, and tigecycline, showing reduced susceptibility to imipenem [3,4]. Moreover, acquired resistance is increasingly reported, due to acquired β-lactamase genes, including narrow-spectrum beta-lactamases (e.g., TEM-1, TEM-2, SHV-1, CARB, inhibitor-resistant TEM IRT), cephalosporinases (e.g., DHA, CMY, ACC-1), extended-spectrum β-lactamase (ESBL) types (TEM, SHV, CTX-M, PER), and carbapenemases (e.g., KPC, OXA-48-like, VIM, IMP, NDM), but also acquired resistance genes to aminoglycosides, quinolones, sulphonamides, and other antimicrobial drug classes [3,4].

Carbapenemases are beta-lactamases with hydrolytic activities against penicillins, cephalosporins, monobactams, and carbapenems, while members of this family may belong to Ambler class A (KPC types), class B metallo-beta-lactamases (VIM, IMP, and NDM types), and class D (OXA-48-like enzymes) [3,4]. OXA-48-like enzymes (OXA-48 and its variants, e.g., OXA-181, OXA-232, OXA-204, OXA-162, and OXA-244) show varying levels of susceptibility against carbapenems and extended-spectrum cephalosporins [5]. They have been spread among different species of *Enterobacterales*, such as *Escherichia coli* and *Klebsiella pneumoniae*, with several reports from different regions worldwide, becoming endemic mostly in Eurasia and Africa [5,6]. However, there are few studies reporting OXA-48-like producers in *P. mirabilis*. The first report of an OXA-48-producing *P. mirabilis* strain (Pm-OXA-48) was described in an outpatient from Gaza, Palestine in 2012 [6], and later on, there were only sporadic cases of OXA-48-like producers mainly from Germany and Spain, co-producing the ESBLs CTX-M-14 and CTX-M-15, respectively [7,8,9].

In *Enterobacterales*, *bla*_OXA-48_ is located between two copies of IS*1999* in variants of the composite transposon Tn*1999*, which is carried on a highly conjugative 63.6-kb IncL plasmid, referred to as pOXA-48 [10]. Seven variants of Tn*1999* (Tn*1999.1* to Tn*1999.6* and invTn*1999.2*) have been described previously among *bla*_OXA-48_ carriers. Most of the Tn*1999* forms have been carried on plasmids. In *P. mirabilis* strain Pm-OXA-48 [7], *bla*_OXA-48_ was also identified on a Tn*1999.1* composite transposon located on an incompatibility group IncL/M type plasmid (pOXA48-PM). Nonetheless, only a few *P. mirabilis* strains described so far have carried the *bla*_OXA-48_ gene on IncL/M plasmids, whereas a high rate of chromosomally integrated *bla*_OXA-48_ has been documented [8,9,10]. The invTn*1999* form seems to be found exclusively in *bla*_OXA-48_-carrying *P. mirabilis* isolates, while its structure may lead to the transposition of the *bla*_OXA-48_ environment via two copies of IS1R [8]. Moreover, *P. mirabilis* strains contain genomic islands carrying MGEs at a high frequency (e.g., transposons and insertion sequences), which are hotspots for the integration of foreign DNA. Thus, it has been suggested that the genetic environment of *P. mirabilis* is highly supportive for chromosomal integration of *bla*_OXA-48_ [8].

In Greece, previous studies have only detected MBLs (VIM-1, VIM-4, and NDM-1 types) among carbapenemase-producing *P. mirabilis* strains [11,12,13,14]. In the present report, we characterize for the first time in Greece an OXA-48-producing *P. mirabilis* strain. It was recovered in the outpatients’ department of a tertiary Greek hospital and belonged to ST135. In addition, whole-genome sequencing (WGS) as well as phylogenetic, phylogenomic, and comparative genomics analyses with previously published *bla*_OXA-48_-harboring *P. mirabilis* genomes were performed to investigate the evolutionary history, the *bla*_OXA-48_ genetic context, and the resistome and virulence factors of Pm strain GR-1.

## 2. Results

### 2.1. Isolation of Bacteria, Antibiotic Susceptibility Testing, and Characterization of β-Lactamases

A swarming Gram-negative rod was recovered on McConkey agar plates from an overnight culture of a urine sample (>100,000 CFU/mL) obtained from a 66-year-old male outpatient who presented during 2023 at the Gennimatas General Hospital, Athens, Greece. The bacterium was identified as *P. mirabilis*, named Pm strain GR-1. No ethnicity or previous history of travel data was available. Antimicrobial susceptibility testing has revealed that Pm strain GR-1 was multidrug-resistant, showing resistance to imipenem (MIC > 4 μg/mL), meropenem (MIC > 8 μg/mL), cefotaxime (MIC > 2 μg/mL), cefepime (MIC > 4 μg/mL), ticarcillin/clavulanic acid (MICs > 16 and 2 μg/mL, respectively), ceftolozane/tazobactam (MICs > 2/2 μg/mL, respectively), ciprofloxacin (MIC > 0.5 μg/mL), levofloxacin (MIC > 1 μg/mL), gentamicin (MIC > 2 μg/mL), tobramycin (MIC > 2 μg/mL), trimethoprim/sulfamethoxazole (MIC > 4 μg/mL), colistin (MIC > 2 μg/mL), and tigecycline (MIC > 0.5 μg/mL). Nonetheless, it was susceptible to ceftazidime/avibactam (MIC < 8/4 μg/mL, respectively) and amikacin (MIC < 8 μg/mL) [Supplementary Table S1]. The lateral flow immunoassay has revealed that Pm GR-1 was positive for OXA-48-like and CTX-M-like ESBL production (Supplementary Figure S1), while the PCR assays were positive for the presence of the *bla*_OXA-48_, *bla*_CTX-M_-like and *bla*_TEM_-like genes.

### 2.2. WGS and In Silico Prediction of MGEs, ARGs, and VFs

WGS was performed for strain Pm GR-1, and the pipelines used and the characteristics of the Pm GR-1 WGS assemblies are shown in Supplementary Tables S2 and S3 and Supplementary Figure S2. Using the OriTfinder tool (available at: https://bioinfo-mml.sjtu.edu.cn/oriTfinder/; accessed on 29 January 2025), an origin of the plasmid transfer site (*oriT*) was not predicted, but it can be provided in trans from other MGEs, whereas a relaxase gene, the gene encoding type IV coupling protein (T4CP), and the gene clusters for bacterial type IV secretion system (T4SS) were identified in the Pm GR-1 genome (Supplementary Table S4). In silico predictions of Mob-type relaxase, MGEs, and ARGs are presented in Table 1. MOB-suite analysis of the long-reads WGS assembly of strain Pm GR-1 (Pm GR-1_11109139608, SRA: SRS25666872) assigned eight of the nine contigs as chromosomal and one contig (contig_0007) as a mobilizable plasmid (primary cluster id: AC864), similar to the *Providencia rettgeri* plasmid p16Pre36-1 (GenBank Accession KX832926), possessing a MobP-type relaxase (Table 1). *bla*_TEM-2_ was located on a plasmid (plasmid cluster id: AC864, contig_0007), whereas *bla*_OXA-48_, *bla*_CTX-M-14_, and *bla*_TEM-1_ β-lactamase genes were carried on chromosomal sequences (Table 1). Strain Pm GR-1 co-carried other ARGs conferring resistance to chloramphenicol (*cat*, *catA1*); trimethoprim (*dfrA17*)*;* sulfamethoxazole (*sul1*); quaternary ammonium compounds (*qacEdelta1);* tetracycline [*tet(J)*]; and aminoglycosides, including gentamicin and tobramycin [*aac(3)-IId*], kanamycin (*aph(3′)-Ia*), streptomycin [*aadA5*, *aph(3″)-Ib*, *aph(6)-Id*], and streptothricin (*sat2*). No acquired resistance genes to quinolones were predicted. Nonetheless, non-sense amino acid substitutions previously associated with quinolone resistance in *P. mirabilis* were identified in the chromosomal DNA gyrase subunits GyrA (S83 → I, AGT → ATT) and GyrB (E466 → D, GAA → GAT) and DNA topoisomerase IV subunits ParC (S80 → I, AGC → ATC) and ParE (S458 → A, TCA → GCA) of strain Pm GR-1 (Supplementary Figure S3). Pm GR-1 also carried resistance genes to stress/heavy metals, including mercury (*merA*, *merC*, *merD*, *merE*, *merP*, *merR*, *merT*) and tellurium (*terD*, *terZ*) (Table 1).

In silico prediction of VFs and protein BLAST (BLASTp, available at: https://blast.ncbi.nlm.nih.gov/Blast.cgi; last assessed 20 February 2025) analyses revealed the presence of several virulence components in the Pm GR-1 genome (Table 2), including the O-antigen locus, the flagella locus, the mannose-resistant Proteus-like (MR/P) fimbriae operon, the urease gene cluster, haemolysin *hpmAB*, and IgA-degrading protease *zapA* genes, which have been associated previously with virulence and pathogenesis traits in *P. mirabilis* [1,2,15,16].

### 2.3. MLST, Virulence ST, Phylogenetic, and Phylogenomic Analyses of Strain Pm GR-1

Strain Pm GR-1 belonged to ST135, as shown by PCR amplification, sequencing of the MLST genes, and in silico prediction of the MLST ST from the WGS assembly. In the PubMLST database (accessed on 1st February 2025), there were 1159 *P. mirabilis* submitted isolates distributed into 470 STs, but only 12 STs were composed of more than 10 isolates (Supplementary Figure S4a). Among them, ST135 was the most prevalent ST (123 isolates), which was diffused into several countries in Europe and Asia and the United States of America during 2012–2022 (Supplementary Figure S4b,c). It should be noted that in the PubMLST database, there is at least one corresponding isolate for each submitted allelic profile/ST, but the collection of isolates does not represent a population sample. Nucleotide BLAST (BLASTn; available at: https://blast.ncbi.nlm.nih.gov/Blast.cgi; accessed on 20 February 2025) searches among *P. mirabilis* genomes (*n* = 1141) showed that 12 genomes carried the *bla*_OXA-48_ gene, but only two of them were of ST135: strain NRZ-54154_b and NRZ-36257 from Germany (Supplementary Figure S5). Notably, MLST analysis has shown that a previously published *P. mirabilis* genome strain AHEPA923 from Greece [13] also belonged to ST135, carrying a *bla*_VIM-78_ gene but no *bla*_OXA-48_ gene.

**Figure 1:**
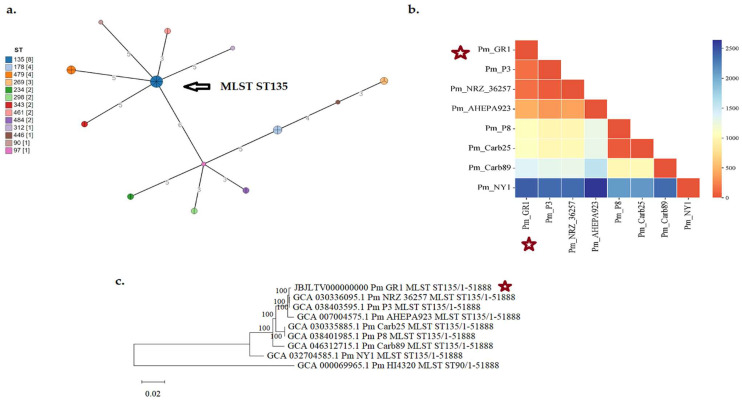
(**a**) Grape Tree phylogenetic analysis of thirty-three studied *P. mirabilis* isolates, (**b**) heatmap matrix of the SNP differences, and (**c**) the core-genome SNP phylogenetic tree of the eight ST135 *P. mirabilis* isolates. The number of isolates comprising each ST are denoted in brackets. Strain Pm GR-1 is denoted with an asterisk.

In order to explore the evolutionary history of strain Pm GR-1, previously published *bla*_OXA-48_-carrying *P. mirabilis* strains were retrieved from public databases (PubMLST and NCBI). Additionally, the genomic sequences of the reference *P. mirabilis* strain HI4320 from the UK (http://bacmap.wishartlab.com/organisms/6770; accessed on 1 January 2025) recovered from a patient with pyelonephritis (16) and *P. mirabilis* strain AHEPA923 from Greece [13] were included in the analyses for comparison. Overall, the final dataset comprised 33 *P. mirabilis* genomes (Supplementary Figure S6, Supplementary Table S5). Grape Tree phylogenetic analysis revealed that ST135 strains were distantly related with the other STs, differing in more than three three alleles of the six MLST loci (Figure 1a). In the final dataset, there were 31 *bla*_OXA-48_-carrying *P. mirabilis* strains distributed into 12 different STs (Figure 1a, Supplementary Table S5). Eight of the isolates were of ST135, which were recovered from Germany (five isolates), Greece (two isolates), and the USA (one isolate). ST135 genomes possessed 31–2381 single-nucleotide polymorphisms (SNPs), using the genome sequence of strain Pm HI4320 as reference (Figure 1b). As shown by the core-genome SNP phylogenetic analysis, Pm strain GR-1 was more closely related to the *bla*_OXA-48_-carrying strains P3 (147 SNP differences) and NRZ36257 (153 SNP differences) from Germany, whereas it was more distantly related (454 SNP differences) to the *bla*_VIM-78_-carrying Pm strain AHEPA923 from Greece (Figure 1b, Supplementary Figure S4b). The core-genome SNP phylogenetic tree of the eight ST135 *P. mirabilis* isolates is shown in Figure 1c.

### 2.4. Comparative Genomics of Strain Pm GR-1

**Figure 2:**
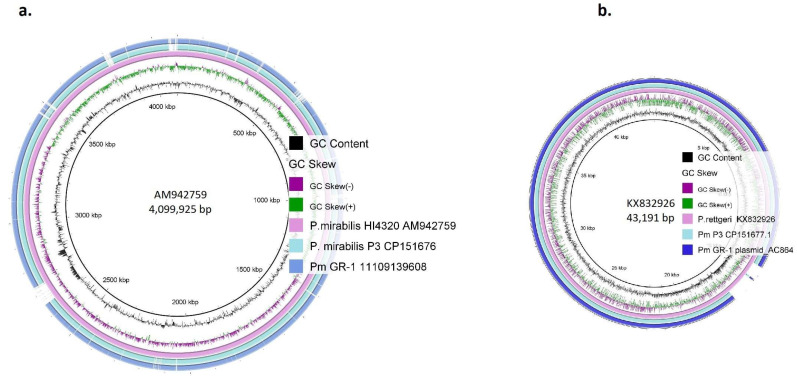
BLASTn comparisons of the (**a**) chromosome and (**b**) plasmid of strain Pm GR-1.

In the genome of strain Pm GR-1, several antimicrobial resistance and virulence genes were located on genomic islands, and comparisons with those present in strains HI4320 and P3 were assessed using the Island Viewer and Island Compare webtools (Supplementary Figure S7, Supplementary Table S6). Out of nine contigs of the long-read WGS assembly of ST135 strain Pm GR-1 (BioSample: SAMN49794400; sample name: Pm GR-1_11109139608; SRA: SRS25666872), eight were highly similar to the chromosomal sequence of ST135 strain P3 from Germany (Accession CP151676) [Figure 2a, Supplementary Figure S8], whereas one contig (contig_0007) was highly similar to one of the two plasmids of strain P3 (Accession CP151677) [Figure 2b, Supplementary Figure S8]. Strain P3 harboured an additional plasmid (Genbank accession no. CP151678), which was not present in strain Pm GR-1.

Comparative genomics revealed high-level similarities in major virulence gene clusters among strains Pm-GR1, P3, and HI4320 (Supplementary Figure S9, Supplementary Table S9). The O-antigen cluster, which was located between the *cpxA* and *secB* genes, showed high similarity with those of strain P3. Strains GR-1, P3, and HI4320 showed high-level similarity in the flagella locus, which is encoded by a single contiguous 54-kb chromosomal sequence containing 50 genes, and the mannose-resistant Proteus-like (MR/P) fimbriae, which are encoded by the *mrp* gene cluster, including two transcripts (*mrpABCDEFGHJ* and *mrp*I) that are transcribed in opposite directions. Moreover, the three *P. mirabilis* strains (GR-1, P3, and HI4320) also possessed highly similar sequences in the urease (*ure)* operon (*ureDABCEFG*), which is under the regulation of UreR. BLASTn searches and alignment of the Pm GR-1 and P3 strains’ genome sequences in the RNA central (the non-coding RNA sequence) database (https://rfam.org; accessed on 20 February 2025) have shown that they both possessed a *cis*-encoded antisense small RNA (5′ureB-sRNA, Rfam Accession no. RF025140, https://rfam.org/family/RF02514, accessed on 20 February 2025), which was highly similar (99%, 282/284 nucleotides) to the 5′ureB-sRNA sequence of *P. mirabilis* strain HI4320 (Accession no. URS00006EF271_584; https://rnacentral.org/rna/URS00006EF271/584, accessed on 20 February 2025).

**Figure 3:**
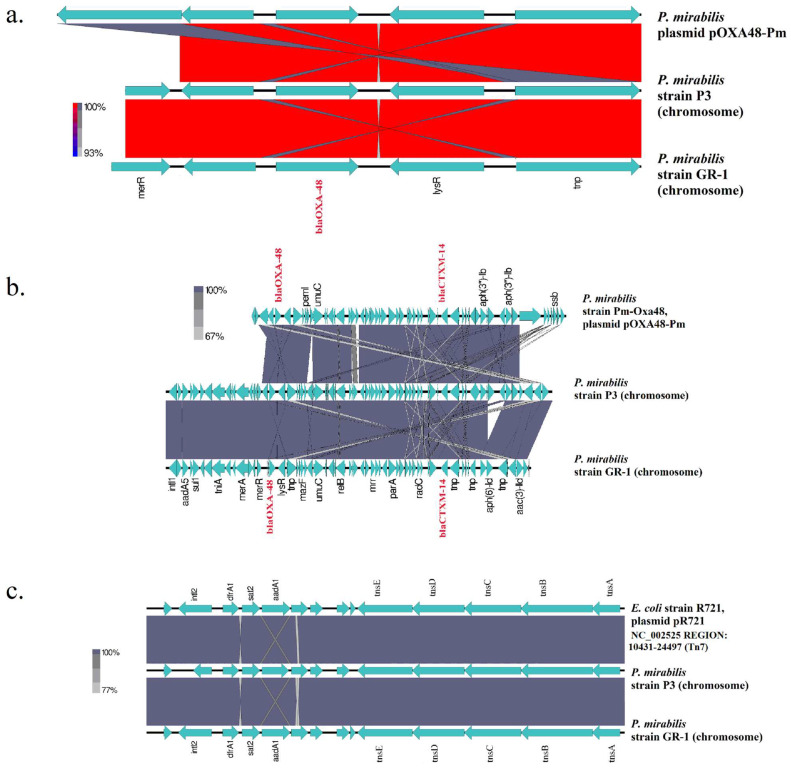
BLASTn comparisons of (**a**,**b**) the *bla*OXA-48-carrying contigs and (**c**) the Tn7 structure of strain Pm GR-1. *bla*_OXA-48_ and *bla*_CTX-M-14_ gene names are denoted in red. The legend bars on the left side of the figures denote the percentage of similarity of the nucleotide sequences.

As shown by comparative analysis (Figure 3a,b), the Pm GR-1 genome assembly carried both *bla*_OXA-48_ located on an *inv*Tn*1999.2* form and *bla*_CTX-M-14_ on a chromosomal genetic structure, which was highly similar to the previously described structure of strain P3 [8]. The predicted Tn7 structure in the Pm GR-1 genome (Table 1) possessed a class 2 integron and the genes encoding the five core proteins (TnsA, TnsB, TnsC, TnsD, and TnsE) required for transposition (Figure 3c). It was identical to the Tn7 structure of *P. mirabilis* strain P3 and *E. coli* plasmid strain R721 (GenBank accession NC_002525 region: 10431–24497).

Comparisons of the genomes of the eight ST135 *P. mirabilis* genomes (seven *bla*_OXA-48_-carrying strains and the *bla*_VIM_-78-carrying strain from Greece) revealed that they carried ARGs to multiple antimicrobial classes, stress resistance genes, and virulence factors (Supplementary Tables S5 and S6). In more detail, they co-carried several beta-lactamase genes, including *bla*_OXA-48_, *bla*_VEB-6_, *bla*_CTX-M-14_, *bla*_CTX-M-15_, *bla*_CTX-M-65_, *bla*_NDM-1_, *bla*_OXA-1_, *bla*_OXA-9_, *bla*_TEM-1A_, *bla*_TEM-1B_, *bla*_TEM-2_, and bleomycin resistance gene (*ble*_MBL_), but also ARGs conferring resistance to rifamycin, aminoglycosides, quinolones, phenicols, trimethoprim, fosfomycin, lincosamides, macrolides, streptogramines, quinolones, sulphonamides, and tetracycline. In addition, strain Pm NY-1 from the USA harboured stress resistance genes to arsenic, copper, silver, the heat shock survival AAA family ATPase *clpK* gene, and the small heat shock protein *sHSP20* gene. In silico prediction of the virulence sequence type (vST) of all studied MLST ST135 *P. mirabilis* strains, including strain Pm GR-1, were assigned to vST138, which was also found in 162 *P. mirabilis* genomes of ST135 (*n* = 118), and 11 other STs (*n* = 107). BURST analysis has shown that these 12 MLST STs clustered into three different clonal complexes [Supplementary Figure S10, Supplementary Table S10].

## 3. Discussion

*P. mirabilis* has an extensive repertoire of antimicrobial resistance mechanisms to last-resort antibiotics, and effective treatment of infections caused by this pathogen maybe challenging [1,2,3]. Due to the varying levels of resistance to carbapenems and piperacillin/tazobactam, accurate detection and treatment of infections caused by OXA-48 producers is difficult, leading to therapeutic failures [4,5,6]. In a recent study, a genomic comparison analysis of 1267 *P. mirabilis* genomes from public databases has shown a widespread resistance observed particularly against beta-lactams, an increasing trend in resistance to carbapenems and quinolones, and a high frequency of urease genes [17]. The total percentage of carbapenem antibiotic resistance genes was as high as 29.5%, including, in decreasing frequency, *bla*_NDM_ (*bla*_NDM-1,5,7_), *bla*_IMP_ (*bla*_IMP-4,6,27_), *bla*_OXA_ (*bla*_OXA-23,48,58_), *bla*_KPC_ (*bla*_KPC-2,3,6_), and *bla*_VIM_ (*bla*_VIM-1,4_). Since the first report of an OXA-48 co-producing CTX-M-14 *P. mirabilis* strain recovered from Gaza, Palestine in 2012, there have been few reports of OXA-48 producers in this species [7,8,9]. Herein, we report the first case in Greece of an OXA-48-co-producing CTX-M-14 *P. mirabilis* strain (named Pm GR-1), which was recovered in 2023 from a urine sample of an outpatient in a General Hospital of Athens, Greece. Furthermore, characterization of strain Pm GR-1 by antimicrobial susceptibility testing, detection of β-lactamases, MLST, WGS, phylogenomics, and comparative genomic analyses was performed.

Antimicrobial susceptibility testing has revealed that Pm strain GR-1 was susceptible to ceftazidime/avibactam and amikacin but resistant to multiple antimicrobial classes, including β-lactams and β-lactam/β-lactamase inhibitor combinations, aminoglycosides, quinolones, trimethoprim/sulfamethoxazole, nitrofurantoin, colistin, tigecycline, and tetracycline. In silico predictions of ARGs were consistent with the antibiogram of Pm strain GR-1, except for quinolones. Nonetheless, chromosomal non-sense mutations were identified in the GyrA, GyrB, ParC, and ParE proteins, which have been associated previously with quinolone resistance. According to the EUCAST Expert Rules v3.3 on *Enterobacterales* (available at: https://www.eucast.org/), aminoglycosides (e.g., gentamicin, tobramycin, and amikacin) should always be used in combination with another active therapy. On occasion, the agents may be used alone for treatment of urinary tract infections with “complicated” bacteria difficult to treat with other agents because of resistance development. Ceftazidime/avibactam is a valuable therapeutic option to treat infections caused by OXA-48-producing *Enterobacterales* in the absence of an MBL, whereas co-resistance mechanisms do not affect the susceptibility of ceftazidime/avibactam, as shown previously [5].

Minimum-spanning tree phylogenetic analysis based on the MLST allelic profiles and core-genome SNP phylogenomic analysis of previously published *P. mirabilis* genomes have revealed that strain Pm GR-1 belonged to ST135, being more related to OXA-48 producers from Germany than one OXA-48 producer from the USA and the VIM-78 producer from Greece. It should be noted that an outbreak of ST135 *bla*_NDM_-carrying *P. mirabilis* strains has also been recently reported in Tunisia, Africa [18]. Thus, the ST135 lineage has the potential for acquisition of various carbapenemase genes, such as *bla*_OXA-48_, *bla*_VIM-78_, and *bla*_NDM_. Comparative genomics showed that strain Pm GR-1 and the phylogenetically related strain P3 from Germany harboured highly similar chromosomal sequences, co-carrying *bla*_OXA-48_ on an invTn*1999.2* transposon form and *bla*_CTX-M-14_, but also a highly similar plasmid. Strain Pm GR-1 also carried several transposon structures (Tn7-like, Tn4352, and composite transposons of the IS4 and an IS6 families) and both class 1 and class 2 integrons. As shown in previous studies, among *P. mirabilis* clinical isolates, class 1 integrons are the predominant type, whereas class 2 integrons are usually carried on Tn7 and its relatives [19,20]. Antibiotic resistance and virulence genes caried on MGEs and trasposons can spread via horizontal gene transfer in other strains and bacterial species, mediating the adaptation of bacteria in clinical settings and natural environments.

Moreover, all eight of the studied ST135 *P. mirabilis* strains belonged to vST138, which was also found among three different clonal complexes of 162 *P. mirabilis* genomes (118 isolates of MLST ST135 and 107 isolates of 11 other STs). Thus, these virulence factors have the potential to diffuse in the *P. mirabilis* population. Comparative genomics of virulence gene clusters among strains Pm GR-1 and P3 from Germany revealed that both strains possessed an identical O-antigen locus. O-antigens are major surface components of the outer membrane and virulence factors of Gram-negative bacteria, and their diversity is associated with the ability of the bacterium to adapt to different hosts and environments [21]. Moreover, based on the O-antigen structural diversity, 80 O-serotypes have been reported in *Proteus* at present. In addition, high-level sequence similarities were observed in the flagella locus, the MR/P fimbriae, and the urease gene cluster of *P. mirabilis* strains GR-1, P3, and HI4320. The flagella locus encodes for a peritrichous flagella, which mediates motility of *P. mirabilis* and enables the bacterium to differentiate from an infectious single-cell rod-shaped form (swimmer cell) to a multi-cell elongated form (swarmer cell) and pass through the urethra to the bladder [1,2,16,22]. Among 17 different fimbriae or pili that mediate adherence to the host mucosal surfaces, the mannose-resistant Proteus-like (MR/P) fimbriae contribute to colonization and biofilm formation by *P. mirabilis* [15,16,22]. In addition, urease is an essential virulence factor for *P. mirabilis* colonization and persistence in UTIs [1,2]. During the course of infection, the production of ammonia by the urease enzyme raises the pH in the local environment of the urinary tract and the subsequent precipitation of polyvalent ions (Mg^2+^ and Ca^2+)^ that are normally soluble in the urine, resulting in stone formation. It is of note that a 5′ureB-sRNA element, which was targeted at the 5′ end of ureB, was identified in the urease gene cluster of strains GR-1, P3, and HI4320. In *Helicobacter pylori*, urease is an essential component of gastric acid acclimation, and the 5′ureB-sRNA was shown previously to downregulate ureAB expression by truncation of the ureAB transcript at a neutral pH [23]. Similarly, the 5′ureB-sRNA may act as an additional control mechanism for the UreR regulator [24] for ureAB expression in *P. mirabilis*.

Finally, there are some limitations considering the present study. We herein analyzed a single ST135 *bla*_OXA-48_-producing *P. mirabilis* strain (Pm GR-1) isolated in a tertiary Greek hospital. However, in previous studies, only outbreaks of *bla*_VIM-1-4_-like carbapenemase-producing *P. mirabilis* have been reported from Greece [11,12,13,14]. Moreover, there was a WGS assembly of a previously published ST135 *P. mirabilis* Greek strain (AHEPA923) which carried *bla*_VIM-78_, suggesting that the international clone ST135 is circulating in Greece. It is also of note that although ST135 is a clone with a worldwide distribution, based on data that we have obtained from the PubMLST database, there are no previous surveillance and epidemiological studies referring to this clone, except from the one describing a Tunisian outbreak of ST135 *bla*_NDM_ producers [18]. Thus, ST135 has the potential to acquire different carbapenemase genes. Lastly, there are scarce data on the prevalence of *bla*_OXA-48_ in *P. mirabilis* from other countries, mainly coming from Germany and Spain. Given that OXA-48-like enzymes often pass undetected, impacting our understanding of their global epidemiology, burden of associated infection, and current treatment outcomes [5], WGS is a valuable tool for detection and characterization of these strains. Nonetheless, further studies are required to assess the prevalence of OXA-48 in this multidrug-resistant and virulent pathogen in Greece and other countries.

## 4. Conclusions

In *P. mirabilis*, *bla*_OXA-48_-carrying strains of diverse MLST STs are sporadically reported, which have caused outbreaks mainly in Germany and Spain. ST135 is one of the most prevalent STs among *P. mirabilis* strains in the PubMLST database with a worldwide distribution, but there are rare reports of ST135 *bla*_OXA-48_-carrying strains. To our knowledge, this is the first report in Greece of a clinical MDR OXA-48-producing *P. mirabilis* strain, named Pm strain GR-1. As shown by WGS, Pm strain GR-1 belonged to the international clone ST135 and possessed several ARGs (including *bla*_OXA-48_ and *bla*_CTX-M-14_ located on the chromosome), virulence genes, genomic islands, MGEs, and a mobilizable *bla*_TEM-2_-carrying plasmid, which may be transferred to other bacteria via horizontal gene transfer. Among 31 *bla*_OXA-48_-carrying strains retrieved from public databases (PubMLST and NCBI) from previous studies, ST135 was identified in strains from Germany and the USA. Moreover, phylogenomic and comparative genomic analyses revealed that Pm strain GR-1 was closely related to *bla*_OXA-48_-carrying strains of ST135 from Germany, carrying highly similar sequences of the chromosomal *bla*_OXA-48_ genetic structures, the O-antigen locus, the flagella locus, the MR/P fimbriae operon, and the urease gene cluster. These findings suggest that MDR and virulent ST135 *bla*_OXA-48_-carrying *P. mirabilis* strains have been emerging in different countries (Germany, Greece, and the USA) in the past few years. The emergence and spread of carbapenemase genes in this multidrug-resistant pathogen is a concern for public health. Thus, accurate detection and characterization of these strains is required for monitoring their prevalence in One Health settings.

## 5. Materials and Methods

### 5.1. Bacterial Identification, Antimicrobial Susceptibility Testing, and Detection of β-Lactamases

The Vitek^®^2 Compact automated system and the Mass Spectrometry Microbial Identification System Vitek^®^ MS PRIME (bioMerieux, Marcy l’Etoile, France) were used for bacterial identification to the species level, according to the instructions of the manufacturer. Antimicrobial susceptibility testing was performed using the Vitek AST N376 and XN10 cards and the broth microdilution method MICRONAUT IVD System (Bruker, Merlin Diagnostika, GmbH, Bornheim, Germany), while the *Escherichia coli* strain ATCC 25922 was used as control, according to the instructions of the manufacturers. Antimicrobial susceptibility testing was conducted according to the EUCAST criteria and S/I/R definitions available at: https://www.eucast.org/ [25]. The NG-Test^®^, (NG Biotech, Guipry-Messac, France) lateral flow immunoassay method was implemented for the detection and differentiation of the five most prevalent families of carbapenemases (KPC, OXA-48, VIM, NDM, and IMP types) and the five major groups of the CTX-M-type ESBLs (CTX-M Groups 1, 2, 8, 9, and 25 types) using the NG-Test^®^ CARBA-5 and the CTX-M MULTI cassettes, respectively, according to the instructions of the manufacturer [26,27]. DNA extraction and screening for the presence of the carbapenemases, CTX-M-type ESBLs, and TEM beta-lactamases by PCR were performed, as previously described [28].

### 5.2. MLST STs, WGS, and Bioinformatic Analyses

Genomic DNA from strain Pm GR-1 was extracted from an overnight culture on Blood Agar and using a Wizard^®^ Genomic DNA Purification Kit (Promega, Madison, WI, USA), according to the manufacturer’s protocol. PCR amplification of the MLST genes (*atpD*, *dnaJ*, *mdh*, *pyrC*, *recA*, *rpoD)* as well as assignment of alleles and STs were performed based on the *Proteus* spp. MLST scheme hosted in the PubMLST database (available at: https://pubmlst.org/organisms/proteus-spp; accessed on 29 January 2025) [29,30,31]. WGS of strain Pm GR-1 was performed using the Ion Torrent platform for short reads and the Nanopore platform for long reads, followed by sequence cleaning and normalisation of the sequencing reads, de novo assembly of high-quality reads, and annotation of the assemblies [32,33,34,35,36]. The WGS pipelines and bioinformatic tools used are described in Supplementary Table S1.

### 5.3. In Silico Prediction of MGEs, ARGs, and VFs

The MOB-Recon and MOB-Typer tools (available at: https://usegalaxy.eu/) of the MOB-suite version 3.1.9 software were used for the characterization of chromosomal and plasmid sequences, reconstruction, extraction, and typing of plasmids from the draft assemblies [37]. The oriTfinder tool (http://bioinfo-mml.sjtu.edu.cn/oriTfinder; accessed on 29 January 2025), was used to explore the presence of conjugative regions of the self-transmissible MGEs: the origin of the transfer site (*oriT*), the relaxase gene, the gene encoding type IV coupling protein (T4CP), and the gene cluster for the bacterial type IV secretion system (T4SS) [38]. Identification of mobile genetic elements and their relation to antimicrobial resistance genes and virulence factors was performed using the Mobile Element finder tool (MGE: https://cge.food.dtu.dk/services/MobileElementFinder/; assessed on 29 January 2025) [39], the AMR Finder Plus in the MicroBIGG-E (Pathogen Detection Microbial Browser for Identification of Genetic and Genomic Elements, https://www.ncbi.nlm.nih.gov/pathogens/microbigge/; accessed on 29 January 2025) database, the CARD database [40] (https://card.mcmaster.ca/; accessed on 29 January 2025), ABRicate, and Integron Finder version 2.0.5 (https://usegalaxy.eu/). Chromosomal mutations associated with quinolone resistance were determined based on the *P. mirabilis* ATCC 29906 nucleotide sequences of the DNA gyrase subunits A (*gyrA*) and B (*gyrB*) and DNA topoisomerase IV subunits ParC (*parC*) and ParE (*parE*); GenBank accession numbers AF397169, AF503506, AF363611, and AF503505, respectively [41]. The virulence allelic profiles and virulence sequence types (vSTs) were assigned based on BLASTn *Proteus* spp. genome comparisons in the PubMLST database. BLASTp comparisons of the annotated predicted proteins of strain Pm GR-1 genome were performed with virulence factors found in the virulence factor database (VFDB: https://www.mgc.ac.cn/VFs/; accessed on 29 January 2025) [42].

### 5.4. Phylogenetic, Phylogenomic, and Comparative Genomic Analyses

The Grape Tree version 2.2 software was used to construct and visualise the phylogenetic tree of *P. mirabilis* strains, using their MLST allelic profiles as input in the MSTree version 2.0 [43]. For phylogenomic analysis, the CSI Phylogeny version 1.4 tool (https://cge.food.dtu.dk/services/CSIPhylogeny/, accessed on 29 January 2025) was used, and the obtained concatenated SNP alignment was incorporated as input in the Molecular Evolutionary Genetic Analysis version 12 (MEGA12) [44] for constructing and visualising the core-genome phylogenetic tree. The evolutionary history of the genome sequences was inferred using the Neighbor-Joining method. The evolutionary distances were computed using the Maximum Composite Likelihood method. The SNP differences were visualized via a heatmap matrix using the SRPlot tool (https://www.bioinformatics.com.cn/en, accessed on 29 January 2025). BLASTn comparisons and construction of the circular maps of the *P. mirabilis* genomes (chromosomes and plasmids) were performed using the BRIG version 0.95 software (http://sourceforge.net/projects/brig, accessed on 29 January 2025) [45], whereas BLAST comparisons and linear alignments were performed using the EasyFig version 2.2.5 (https://mjsull.github.io/Easyfig/, accessed on 29 January 2025) software [46]. Genomic islands were identified and compared using the Island Viewer version 4 (https://www.pathogenomics.sfu.ca/islandviewer/, accessed on 29 January 2025) and Island Compare version 1.12.2.5 (https://islandcompare.ca/, accessed on 29 January 2025) webtools [47].

## Figures and Tables

**Table 1:** In silico predictions of chromosomal and plasmid sequences, Mob-type relaxase, MGEs, and ARGs in strain Pm GR-1.

Pm GR-1 11109139608_(Nanopore)	Molecule Type	Size (bp)	GC (%)	MGEs	ARGs
contig_0001	chromosome	1,905,607	39.72	Tn7, IS26	*aadA5*, *cat*, *dfrA17*, *qacEdelta1*, *sul1*, *merA*, *merC*, *merD*, *merE*, *merP*, *merR*, *merT*
contig_0002	chromosome	915,722	40.26	ISVsa5, Tn4352, IS26, IS629, IS5, cn_2401_ISVsa5, cn_3556_IS26	*bla*_OXA-48_, *bla*_CTX-M-14_, *bla*_TEM-1_, *aac(3)-IId*, *aadA5*, *aph(3′)-Ia*, *aph(3″)-Ib*, *aph(6)-Id*, *catA1*, *dfrA17*, *qacEdelta1*, *sat2*, *sul1*, *tet(J)*, *merA*, *merC*, *merD*, *merE*, *merP*, *merR*, *merT*, *terD*, *terZ*
contig_0003	chromosome	820,194	38.30		
contig_0004	chromosome	308,558	37.36		
contig_0005	chromosome	295,079	38.20		
contig_0006	chromosome	45,133	40.69		
contig_0007	plasmid AC864 *	40,918	36.21		*bla*_TEM-2_
contig_0008	chromosome	5453	39.57		
contig_0009	chromosome	4956	38.03		

* relaxase_type: MobP (Accession: CP021855_00025), mash_nearest_neighbor accession: KX832926 *Providencia rettgeri*, mash_neighbor_distance: 0.0025468.

**Table 2:** Major virulence factors in strain Pm GR-1.

Virulence Factors	Length (bp)	Pm GR-1_11109139608 Contig	Coordinates
vST138 allelic profile			
*cheB* (allele 1)	1053	contig_0003	510,332–511,384
*cheY* (allele 1)	390	contig_0003	509,883–510,272
*flgG* (allele 52)	783	contig_0003	497,099–497,881
*flgH* (allele 46)	744	contig_0003	496,293–497,036
*fliI* (allele 79)	1374	contig_0003	483,487–484,860
*flip* (allele 56)	771	contig_0003	489,299–490,069
*flN* (allele 1)	411	contig_0003	488,439–488,849
*gmhA* (allele 46)	579	contig_0001	607,440–608,018
*KdsA* (allele 66)	855	contig_0004	134,233–135,087
*lpxC* (allele 57)	918	contig_0005	118,044–118,961
*lpxD* (allele 55)	1029	contig_0002	161,072–162,100
*luxS* (allele-43)	516	contig_0001	573,194–573,709
haemolysin genes			
*hpmA*, *B*	4733	contig_0005	126,318–132,770
*zapA*	1475	contig_0001	772,637–774,112
O-antigen locus	19,356	contig_0001	1,638,693–1,658,049
flagella locus	53,161	contig_0003	468,664–521,825
MR/P fimbriae operon	9500	contig_0001	784,087–793,586
urease gene cluster	4195	contig_0001	1,121,504–1,125,699
hydrogenase system *(hybOABCDE*)	5081	contig_0001	1,028,369–1,034,350
molybdate-binding Protein (*modA*)	770	contig_0003	410,098–410,868
sigma factor RpoE (*rpoE)*	548	contig_0001	1,098,303–1,098,851
polyphosphate kinase 1 (*ppk1*)	2081	contig_0003	413,425–415,506

## Data Availability

This Whole Genome Shotgun project has been deposited at DDBJ/ENA/GenBank under the accession Bioproject accession no. PRJNA1191495, Biosample accession no. SAMN45082766, WGS accession no. JBJLTV000000000, the short-reads WGS assembly SRA accession no. SRS23384379 and the long-reads WGS assembly (Pm GR-1_11109139608) SRA accession no. SRS25666872.

## Supplementary Materials

The following supporting information can be downloaded at: https://www.mdpi.com/article/10.3390/antibiotics14080750/s1, Figure S1. OXA-48-like and CTX-M-like β-lactamase detection by the lateral flow immunoassay in strain Pm GR-1. Figure S2. Coverage plot and coverage histogram obtained by the QualiMap BamQC report of the Pm GR-1_11109139608 long-read (Nanopore) genome sequence. Figure S3. Protein alignments of the GyrA, GyrB, ParC, and ParE sequences of strain Pm GR-1 and the wild-type (quinolone-susceptible) *P. mirabilis* strain ATCC 29906. Figure S4. (a) Distribution of *P. mirabilis* isolates from the PubMLST database into MLST STs, and (b) geographical distribution of ST135 and (c) the timeline of isolation of ST135 isolates. Figure S5. (a) SplitsTree analysis and (b) SNP differences of the genomic sequences of strain Pm GR-1 and 12 *bla*_OXA-48_-carrying *P. mirabilis* strains from the PubMLST database. The genomic sequence of *P. mirabilis* strain HI4320 was used as reference. Figure S6. Geographical distribution of (a) MLST STs and (b) β-lactamase content of 33 studied *P. mirabilis* strains. Figure S7. Comparative genomics of predicted genomic islands in *P. mirabilis* strains GR-1, P3, and HI4320. Figure S8. BLASTn comparisons of the long-read WGS assembly Pm GR-1_11109139608 (SRA: SRS25666872) contigs with the chromosome (accession no. CP151676) and the plasmid (accession no. CP151677) of strain P3. Figure S9. Comparative genomics of the: (a) O-antigen gene cluster, (b) flagella locus, (c) MR/P fimbriae operon, and (d) urease gene cluster of strain Pm GR-1. Figure S10. Clonal complexes identified among 162 genomes of virulence type vST138 *P. mirabilis* in the PubMLST database. Table S1. Antimicrobial susceptibility testing of strain Pm GR-1. Table S2. QualiMap BamQC report parameters of the Pm GR-1_11109139608 long-reads (Nanopore) genome sequence. Table S3. WGS pipelines and characteristics of WGS assemblies of strain Pm GR-1. Table S4. In silico predictions using oriTfinder of the *oriT* gene, relaxase gene, T4CP, and the T4SS gene clusters in the Pm GR-1 genome. Table S5. β-lactamase content, MLST STs and country of isolation of 33 studied *P. mirabilis* genomes. Table S6. In silico prediction of genomic islands (GIs) in strain Pm GR-1. Table S7. In silico predictions of ARGs among the eight studied ST135 *P. mirabilis* isolates. Table S8. In silico predictions of stress/heavy metal resistance genes among the eight studied ST135 *P. mirabilis* isolates. Table S9. BLASTp results of the predicted proteins of strain Pm GR-1 genome and the VFDB dataset. The proteins showing >60% similarity are shown. Table S10. BURST analysis of virulence type vST138 *P. mirabilis* (*n* = 162) in the PubMLST database.

## Abbreviations

The following abbreviations are used in this manuscript:

ARG	antimicrobial resistance gene
GI	genomic island
MDR	multidrug-resistant
MGE	mobile genetic element
MLST	multilocus-sequence typing
SNP	single-nucleotide polymorphism
ST	sequence type
VF	virulence factor
