# Supplementary material for: Genomic Insights of Emerging Multidrug-Resistant OXA-48-Producing ST135 Proteus mirabilis
Source: Antibiotics (Basel). 2025 Jul 25;14(8):750. doi: 10.3390/antibiotics14080750 (PMC12383092; doi:10.3390/antibiotics14080750)
Supplement: Supplementary file 1 [file antibiotics-14-00750-s001.zip › antibiotics-3726930-supplementary.pdf]

SUPPLEMENTARY MATERIAL

Supplementary Figures:

Figure S1. OXA-48-like and CTX-M-like β-lactamase detection by the lateral flow immunoassay in strain Pm GR-1.
Figure S2. Coverage plot and coverage histogram obtained by the QualiMap BamQC report of the Pm GR-1_11109139608 long-read (Nanopore) genome sequence.
Figure S3. Protein alignments of the GyrA, GyrB, ParC and ParE sequences of strain Pm GR-1 and the wild-type (quinolone-susceptible) *P. mirabilis* strain ATCC 29906.
Figure S4. (a) Distribution of *P. mirabilis* isolates from the PubMLST database into MLST STs, and (b) geographical distribution of ST135 and (c) the timeline of isolation of ST135 isolates.
Figure S5. (a) SplitsTree analysis and (b) SNP differences of the genomic sequences of strain Pm GR-1 and 12 bla_{OXA-48}-carrying *P. mirabilis* strains from the PubMLST database. The genomic sequence of *P. mirabilis* strain HI4320 was used as reference.
Figure S6. Geographical distribution of (a) MLST STs and (b) β-lactamase content of 33 studied *P. mirabilis* strains.
Figure S7. Comparative genomics of predicted genomic islands in *P. mirabilis* strains GR-1, P3 and HI4320.
Figure S8. BLASTn comparisons of the long-reads WGS assembly Pm GR-1_11109139608 (SRA: SRS25666872) contigs with the chromosome (accession no. CP151676) and the plasmid (accession no. CP151677) of strain P3.
Figure S9. Comparative genomics of the: (a) O-antigen gene cluster, (b) flagella locus, (c) MR/P fimbriae operon, and (d) urease gene cluster of strain Pm GR-1.
Figure S10. Clonal complexes identified among 162 genomes of virulence type vST138 *P. mirabilis* in the PubMLST database.

Supplementary Tables:
Table S1. Antimicrobial susceptibility testing of strain Pm GR-1.
Table S2. QualiMap BamQC report parameters of the Pm GR-1_11109139608 long-reads (Nanopore) genome sequence.
Table S3. WGS pipelines and characteristics of WGS assemblies of strain Pm GR-1.
Table S4. *In silico* predictions using oriTfinder of the *oriT* gene, relaxase gene, T4CP and the T4SS gene clusters in the Pm GR- genome.
Table S5. β-lactamase content, MLST STs and country of isolation of 33 studied *P. mirabilis* genomes.
Table S6. *In silico* prediction of genomic islands (GIs) in strain Pm GR-1.
Table S7. *In silico* predictions of ARGs among the eight studied ST135 *P. mirabilis* isolates.
Table S8. *In silico* predictions of stress/heavy metal resistance genes among the eight studied ST135 *P. mirabilis* isolates.
Table S9. BLASTp results of the predicted proteins of strain Pm GR-1 genome and the VFDB dataset. The proteins showing >60% similarity are shown.
Table S10. BURST analysis of virulence type vST138 *P. mirabilis* (n=162) in the PubMLST database.

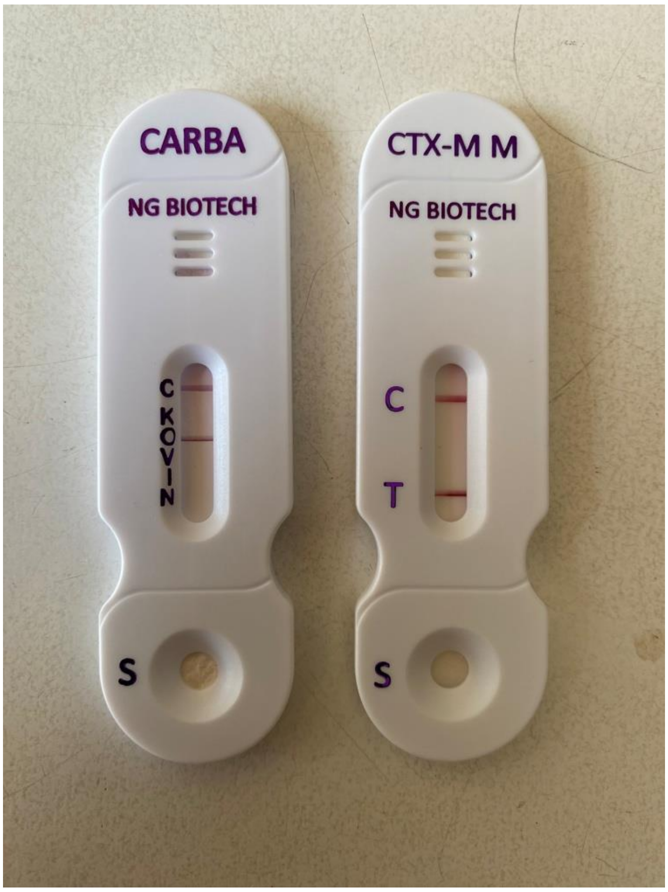

Figure S1. OXA-48-like and CTX-M-like β-lactamase detection by the lateral flow immunoassay in strain Pm GR-1.

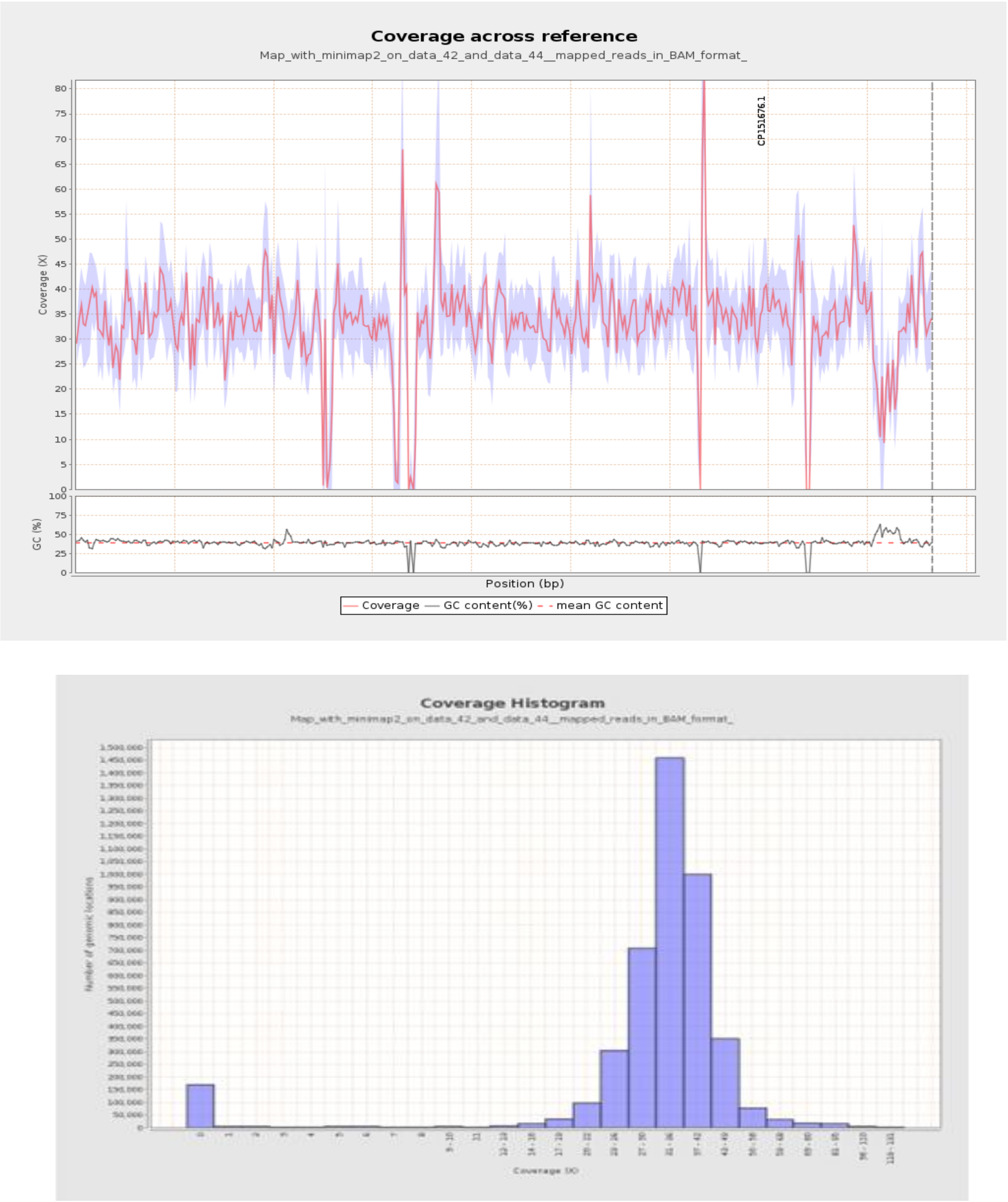

Figure S2. Coverage plot and coverage histogram obtained by the QualiMap BamQC report of the Pm GR-1_11109139608 long-read (Nanopore) genome sequence. The Pm GR-1_11109139608 long-read (Nanopore) genome sequence was aligned with the chromosomal genome sequence of strain P3 (Accession CP151676) using the minimap2 tool, and the quality of the aligned reads were evaluated by the QualiMap BamQC tool (available at: https://usegalaxy.eu/).

A. GyrA

```
                                                                                   8
                                                                                   3
#AF397169.1_Proteus mirabilis_ATCC29906      LKPVHRRVLF AMNVLGNDWN KPYKKSARVV GDVIGKYHPH GDSAVYETIV
#Pm_GR-1_gyrA                                .......... .......... .......... .......... ..I.......

#Pm_ATCC29906_Proteus mirabilis_ATCC29906    RLAQPFSMRY MLVDGQGNFG SVDGDSAAAM RYTEVRMAKI AHELLAD
#Pm_GR-1                                     .......... .......... .......... .......... .......
```

B. GyrB

```
#AF503506.1_Proteus mirabilis_ATCC29906      LIAVISVKVP DPKFSSQTKD KLVSSEVKTA VETLMNEKLV EYLLENPTDA
#Pm_GR-1                                     .......... .......... .......... .......... ..........

#AF503506.1_Proteus mirabilis_ ATCC29906     KIVVGKIIDA ARAREAARKA REMTRRKGAL DLGGLPGKLA DCSERDPAFS
#Pm_GR-1                                     .......... .......... .......... .......... ..........

                                                                                            4
                                                                                            6
                                                                                            6
#AF503506.1_Proteus mirabilis_ATCC29906      ELYLVEGDSA GGSAKQGRNR KTQAILPLKG KILNVEKARF DKMLASQEVA
#Pm_GR-1                                     .......... .......... .......... .......... .......D..

#AF503506.1_Proteus mirabilis_ATCC29906      TLITALGCGI GRDEYNPDKL RYHSI
#Pm_GR-1                                     .......... .......... .....
```

C. ParC

```
                                                                                 8
                                                                                 0
#AF363611.1_Proteus mirabilis_ATCC29906    LKPVQRRIVY AMSELGLNSS AKFKKSARTV GDVLGKYHPH GDSACYEAMV
#Pm_GR-1                                   .......... .......... .......... .......... ..I.......

#AF363611.1_Proteus mirabilis_ATCC29906    LMAQPFSYRY PLIDGQGNWG APDDPKSFAA MRYTESRLSK YSQILLSELG
#Pm_GR-1_parC_contig_0002_249287-249634    .......... .......... .......... .......... ..........

#AF363611.1_Proteus mirabilis _ATCC_29906 HGTVDWIPNF DGTLQE
#Pm_GR-1                                   .......... ......
```

D. ParE

```
#AF503505.1_Proteus mirabilis_ATCC29906 GQTKERLSSR QTSAFVASAV KNAFSLWLNQ NVQVGELLAE MAISSAQRRM
#Pm_GR-1                                .......... .......... .......... .......... ..........

#AF503505.1_Proteus mirabilis_ATCC29906 RAAKKVVRKK LTSGPALPGK LADCTSQDLR YTELFLVEGD SAGGSAKQAR
#Pm_GR-1                                .......... .......... .......... .......... ..........

                                                                   4
                                                                   5
                                                                   8
#AF503505.1_Proteus mirabilis_ATCC29906 DREYQAIMPL RGKILNTWEV SSDEVLASQE VHDISVAIGM DPDSDDLSQL
#Pm_GR-1                                .......... .......... .......A.. .......... ..........

#AF503505.1_Proteus_mirabilis_ATCC29906 RYGKICIL
#Pm_GR-1                                ........
```

Figure S3. Protein alignments of the GyrA, GyrB, ParC and ParE sequences of strain Pm GR-1 and the wild-type (quinolone-susceptible) *P. mirabilis* strain ATCC 29906.

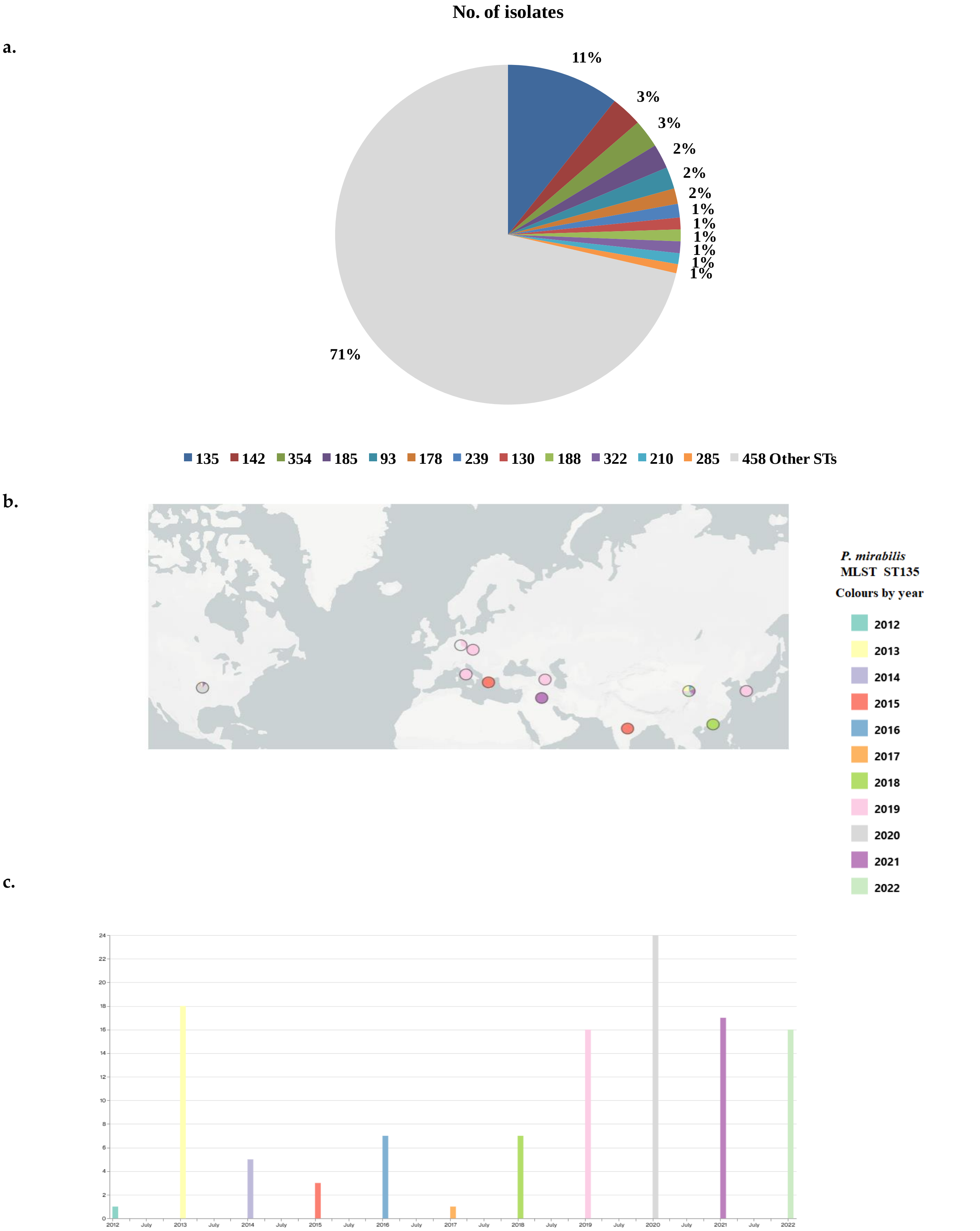

Figure S4. (a) Distribution of *P. mirabilis* from the PubMLST database into MLST STs, and (b) geographical distribution and (c) the timeline of isolation of ST135 isolates.

a.

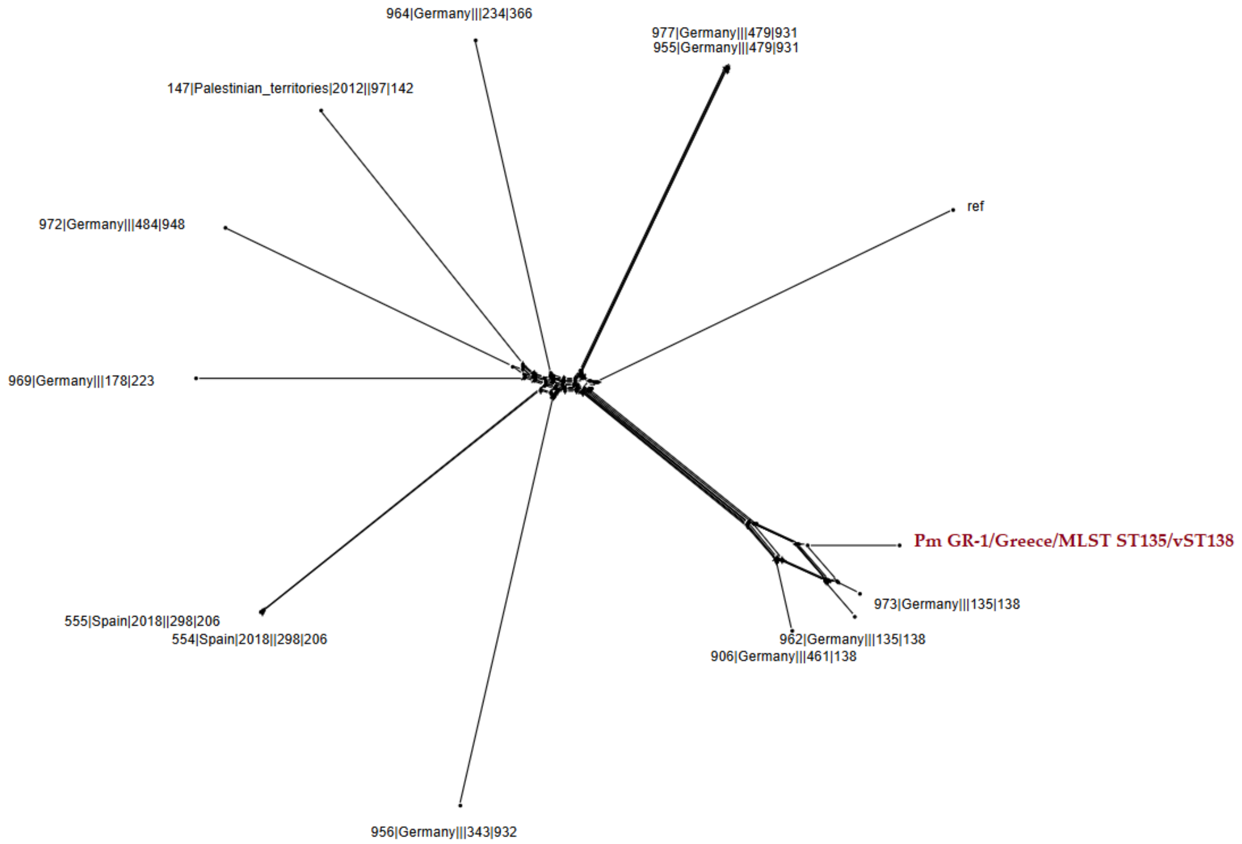

b.

	Pm GR-1|Greece|2023| |135|138	reference genome	147|Palestinian_territories|2012| |97|142	554|Spain|2018| |298|206	555|Spain|2018| |298|206	906|Germany| | |461|138	955|Germany| | |479|931	956|Germany| | |343|932	962|Germany| | |135|138	964|Germany| | |234|366	969|Germany| | |178|223	972|Germany| | |484|948	973|Germany| | |135|138	977|Germany| | |479|931
**Pm GR-1|Greece|2023| |135|138**	0													
**reference genome**	2915	0												
**147|Palestinian_territories|2012||97|142**	2856	3028	0											
**554|Spain|2018||298|206**	2891	3044	2689	0										
**555|Spain|2018||298|206**	2890	3043	2687	12	0									
**906|Germany|||461|138**	1036	2801	2779	2788	2787	0								
**955|Germany|||479|931**	2776	2832	2747	2797	2795	2608	0							
**956|Germany|||343|932**	3072	3254	3002	2978	2977	2933	3026	0						
**962|Germany|||135|138**	741	2858	2838	2873	2872	646	2707	3050	0					
**964|Germany|||234|366**	2817	2981	2633	2696	2695	2705	2701	3041	2796	0				
**969|Germany|||178|223**	2839	3069	2575	2692	2691	2604	2759	2959	2797	2687	0			
**972|Germany|||484|948**	2861	3069	2480	2732	2730	2724	2784	2987	2798	2675	2502	0		
**973|Germany|||135|138**	608	2775	2805	2848	2847	627	2653	3031	306	2735	2787	2781	0	
**977|Germany|||479|931**	2766	2839	2746	2801	2799	2608	26	3026	2711	2684	2773	2796	2652	0

Figure S5. (a) SplitsTree analysis and (b) SNP differences of the genomic sequences of strain Pm GR-1 and 12 *bla*OXA-48-carrying *P. mirabilis* strains from the PubMLST database. The genomic sequence of *P. mirabilis* strain HI4320 was used as reference.

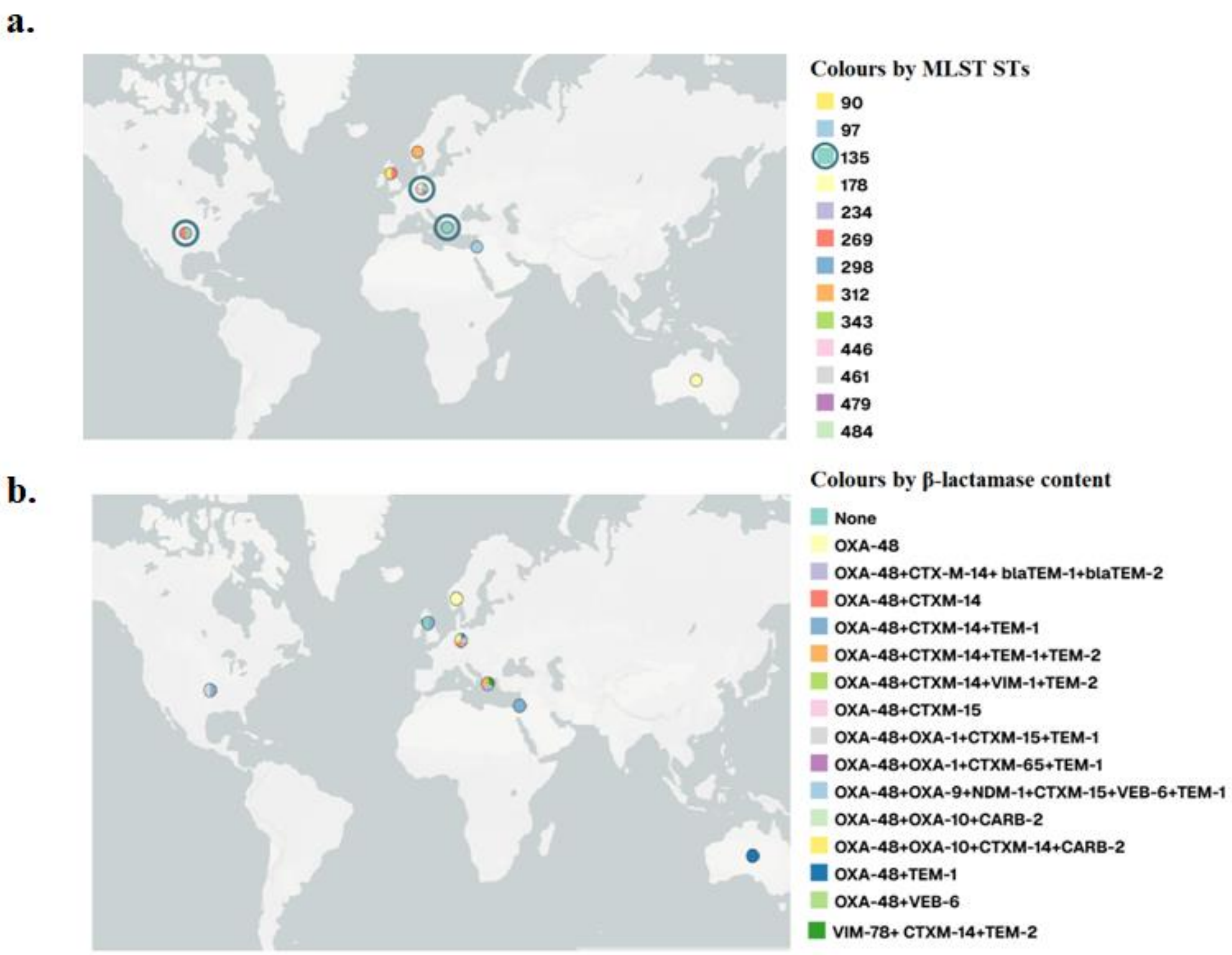

Figure S6. Geographical distribution of (a) MLST STs and (b) β-lactamase content of 33 studied *P. mirabilis* strains.

a.

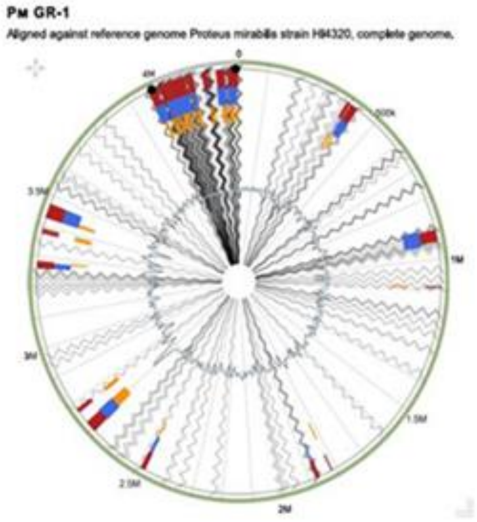

b.

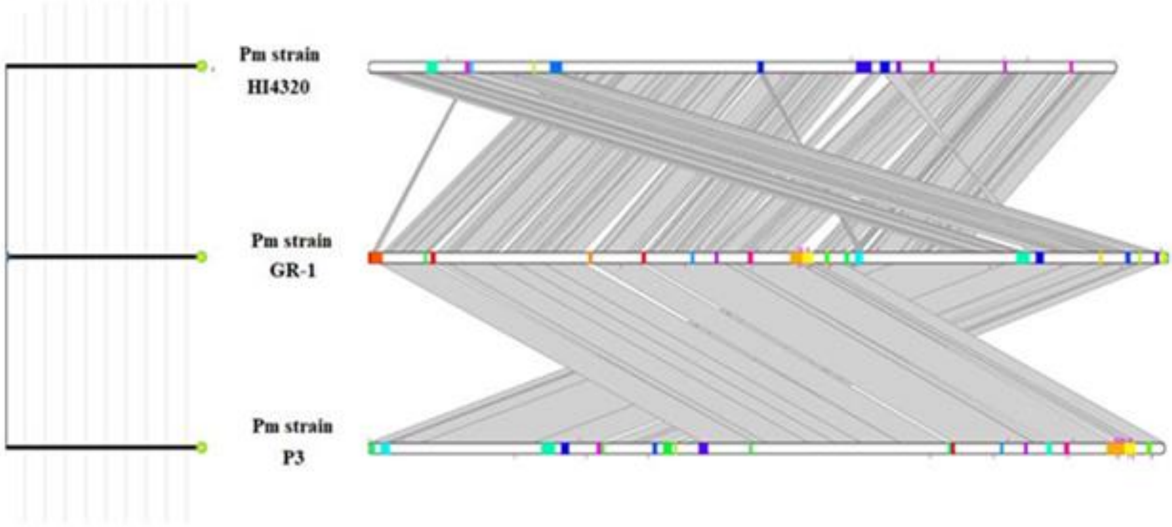

Figure S7. Comparative genomics of predicted genomic islands in *P. mirabilis* strains GR-1, P3 and HI4320.

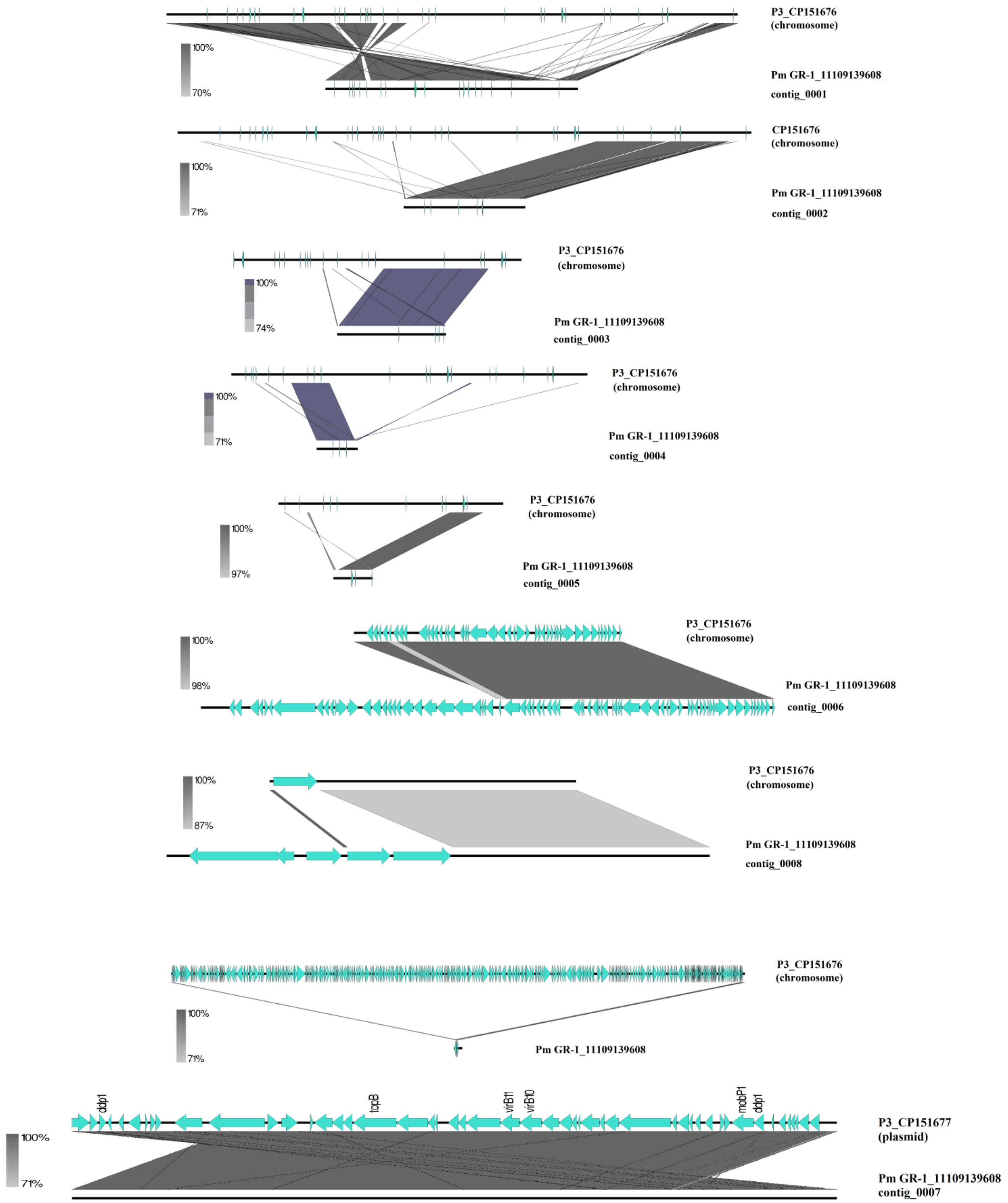

Figure S8. BLASTn comparisons of the long-read WGS assembly Pm GR-1_11109139608 (SRA: SRS25666872) contigs with the chromosome (accession no. CP151676) and the plasmid (accession no. CP151677.1) of strain P3.

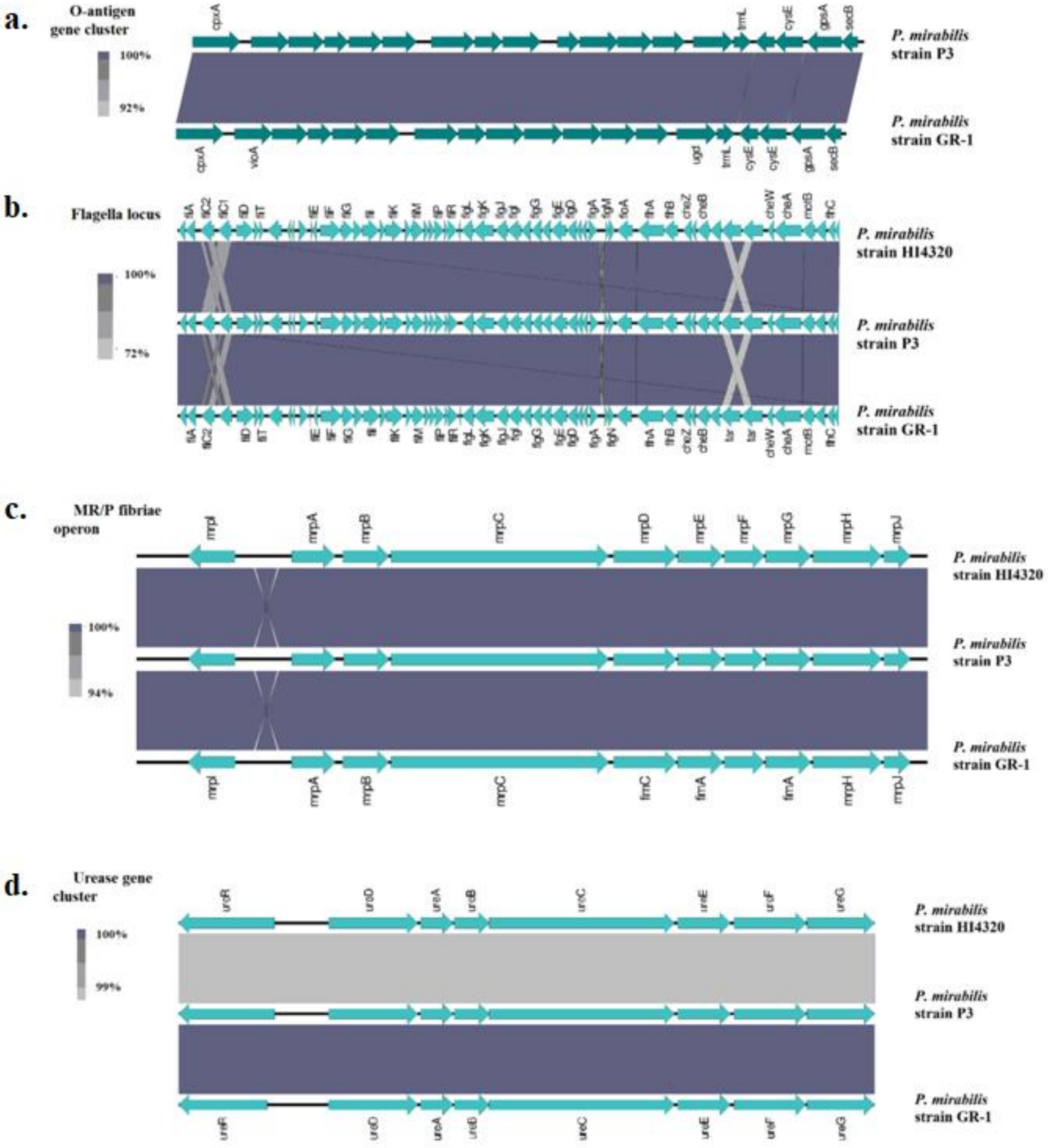

Figure S9. Comparative genomics of the: (a) O-antigen gene cluster, (b) flagella locus, (c) MR/P fimbriae operon, and (d) urease gene cluster of strain Pm GR-1. The bars on the left denote the percentage of similarity of the nucleotide sequences.

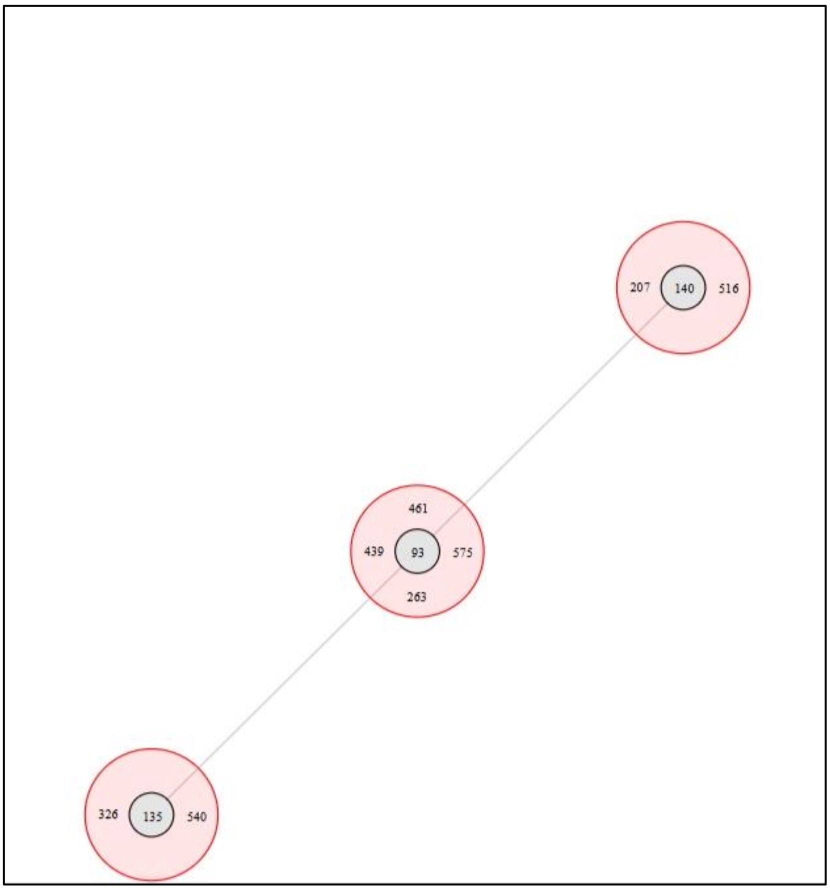

Figure S10. Clonal complexes identified among 162 genomes of virulence type vST138 *P. miralis* in the PubMLST database.

Table S1. Antimicrobial susceptibility testing of strain Pm GR-1.

Antimicrobial Agent	EUCAST MIC Breakpoints (μg/ml) [a]		MIC (μg/ml)	Interpretation
	S ≤	R >		
**Vitek®2 Compact AST System**				
Ampicillin/Sulbactam	8	8	>16/8	R
Ampicillin	8	8	>16	R
Cefepime	1	4	>16	R
Cefotaxime	1	2	>32	R
Ceftazidime	1	4	>16	R
Cefuroxime (iv)	0.001	8	>16	R
Cefuroxime oral (uncomplicated UTI only)	8	8	>16	R
Ciprofloxacin	0.06	0.06	>2	R
Ertapenem	0.5	0.5	1	R
Fosfomycin i.v	*P. mirabilis* ECOFF= 8[b]		>64	R
Gentamicin (infections originating from the urinary tract)	8	8	>8	R
Imipenem (*Morganellaceae*)	0.001	4	8	R
Levofloxacin	0.5	1	>4	R
Meropenem (indications other than meningitis)	2	8	>8	R
Piperacillin	8	8	>64	R
Piperacillin/ Tazobactam	8	8	>64	R
Tobramycin (infections originating from the urinary tract)	2	2	>8	R
Trimethoprime/ Sulfamethoxazole	2	4	>4/76	R
**MICRONAUT IVD System**				
Amikacin (infections originating from the urinary tract)	8	8	< 2	S
Colistin	(2) [b]	(2) [b]	>16	R
Tigecycline	0.5	0.5	>1	R
Ceftolozane/Tazobactam	2	2	0.5/4	R
Ceftazidime/Avibactam	8	8	<0.5/4	S

[a] https://www.eucast.org/fileadmin/src/media/PDFs/EUCAST_files/Breakpoint_tables/v_15.0_Breakpoint_Tables.pdf

[b] https://www.eucast.org/fileadmin/src/media/PDFs/EUCAST_files/Guidance_documents/Breakpoints_in_brackets.pdf
For these agents, the tradition is to use them in combination with other effective measures, often another active agent, to compensate for the inherent inadequacy of the agent. On occasion, the agents may be used alone when they are significantly concentrated at the site of infection. For instance, treatment of urinary tract infections with "complicated" bacteria difficult to treat with other agents because of resistance development. Isolates with resistance (MIC above or zone diameter below the R breakpoint in bracket) can be reported R (resistant) but reporting S or I should be avoided and if considered necessary, there should be a comment to explain the need for adjunctive measures as mentioned above.

Table S2. QualiMap BamQC report parameters of the Pm GR-1_11109139608 long-read (Nanopore) genome sequence.

**Chromosome stats***	
**Reference Name**	**CP151676.1**
Length	4,327,476
Mapped	145207865
**Globals**	
Reference size	4,327,476
Number of reads	36,648
Mapped reads	35,792 / 97.66%
Unmapped reads	856 / 2.34%
Mapped paired reads	0 / 0%
Secondary alignments	1,079
Supplementary alignments	775 / 2.11%
Read min/max/mean length	0 / 50,687 / 4,142.4
Overlapping read pairs	0 / 0%
Duplicated reads (flagged)	0 / 0%
Duplicated reads (estimated)	2,231 / 6.09%
Duplication rate	2.65%
Clipped reads	20,126 / 54.92%
**ACGT Content**	
Number/percentage of A's	44,056,176 / 30.56%
Number/percentage of C's	28,220,945 / 19.58%
Number/percentage of T's	44,017,695 / 30.54%
Number/percentage of G's	27,851,323 / 19.32%
Number/percentage of N's	0 / 0%
GC Percentage	38.9%
**Coverage**	
Mean	33.5549
Standard Deviation	11.054
**Mapping Quality**	
Mean Mapping Quality	57.89
**Mismatches and indels**	
General error rate	1.64%
Insertions	234,818
Mapped reads with at least one insertion	89.87%
Deletions	346,314
Mapped reads with at least one deletion	94.26%
Homopolymer indels	34.6%

* The Pm GR-1_11109139608 long-read (Nanopore) genome sequence was aligned with the chromosomal genome sequence of strain P3 (Accession CP151676.1) using the minimap2 tool, and the quality of the aligned reads were evaluated by the QualiMap BamQC tool (available at: https://usegalaxy.eu/).

Table S3. WGS pipelines and characteristics of WGS assemblies of strain Pm GR-1.

**Assembly and annotation characteristics**	**Genome assembly ASM5130863v1 JBJLTV010000000**	**Pm GR-1_11109139608; SRA: SRS25666872**	**Unicycler**
**Sequencing technology**	Ion Torrent (short-reads)	Nanopore (long-reads)	Ion Torrent + Nanopore (Hybrid assembly)
**QUAST statistics without reference***			
# contigs	147	9	15
# contigs (>= 0 bp)	276	9	15
# contigs (>= 1000 bp)	115	9	14
Largest contig	207,438	1,905,607	1,752,173
Total length	4,174,213	4,341,620	4,301,359
Total length (>= 0 bp)	4,209,707	4,341,620	4,301,359
Total length (>= 1000 bp)	4,151,617	4,341,620	4,300,648
N50	100,815	915,722	943,177
N90	19,059	308,558	108,288
auN	96,793	1,227,341	1,021,232
L50	15	2	2
L90	54	4	7
GC (%)	39	39.27	39.19
Per base quality			
# N'sper 100 kbp	38.33	0	0
# N's	1,600	0	0
**Annotation**			
Annotation Pipeline	NCBI PGAP	Bakta: v1.8.2	Not done
CDS (total)	3,989	4,049	Not done
tRNAs	51	86	Not done
rRNAs	10	22	Not done
ncRNAs	4	51	Not done
CRISPR arrays	-	3	Not done
pseudogenes	403	21	Not done

Table S4. *In silico* predictions using oriTfinder of the *oriT* gene, relaxase gene, T4CP and the T4SS gene clusters in the Pm GR-1 gene genome.

Type	Location	Locus tag/Genename
Origin of the plasmid transfer site *(oriT)* gene	-	-
Relaxase gene	632,709- 634,301	LIMFOD_03095
Type IV coupling protein (T4CP)	255,426- 259,283<br>661,695- 663,842	LIMFOD_01205<br>LIMFOD_03275
Auxiliaryprotein	-	-
Bacterial type IV secretion system (T4SS) gene cluster	644,825- 667,540	LIMFOD_03185; LIMFOD_03190; LIMFOD_03195; LIMFOD_03200; LIMFOD_03205; LIMFOD_03215; LIMFOD_03220; LIMFOD_03230; LIMFOD_03235; LIMFOD_03240; LIMFOD_03245; LIMFOD_03250; LIMFOD_03255; LIMFOD_03260; LIMFOD_03270; LIMFOD_03285; LIMFOD_03290; LIMFOD_03295; LIMFOD_03305

Table S5. β-lactamase, content, MLST STs and country of isolation of 33 studied *P. mirabilis* genomes.

GenBank Accession	Strain;<br>Sample aliases	β-lactamases	MLST ST	Country
GCA_000069965.1	HI4320	None	90	GreatBritain
GCA_000770765.1	Pm-Oxa48	OXA-48+CTXM-14+TEM-1	97	Palestine
JBJLTV000000000	Pm GR-1	OXA-48+CTXM-14+TEM-1+TEM-2	135	Greece
GCA_007004575.1	AHEPA923	VIM-78+ CTXM-14+TEM-2	135	Greece
GCA_030335885.1	Carb-25; NRZ-54154_b	OXA-48+OXA-1+CTXM-65+TEM-1	135	Germany
GCA_046312715.1	Carb-89; NRZ-85578	OXA-48+OXA-9+NDM-1+CTXM-15<br>+VEB-6+TEM-1	135	Germany
GCA_030336095_1	NRZ-36257	OXA-48+CTXM-14+TEM-2	135	Germany
GCA_038403595.1	P3; PM-OXA-03	OXA-48+CTXM-14+TEM-1+TEM-2	135	Germany
GCA_038401985.1;	P8; PM-OXA-08	OXA-48+OXA-1+CTXM-65+TEM-1	135	Germany
JBJLTV000000000	Pm GR-1	OXA-48+CTXM-14+TEM-1+TEM-2	135	Greece
GCA_032704585.2	NY-1; 2023HL-00813	OXA-48+OXA-1+CTXM-15+TEM-1	135	USA
GCA_038070545.1	P10; PM-OXA-10	OXA-48+OXA-10+CTXM-14+CARB-2	178	Germany
GCA_016065595	AUS1	OXA-48+TEM-1	178	Australia
GCA_030336015.1	Carb-18; NRZ-40358	OXA-48+OXA-10+CTXM-14+CARB-2	178	Germany
GCA_046311375.1	Carb-64; NRZ-75365	OXA-48+OXA-10+CARB-2	178	Germany
GCA_030335925.1	Carb-27; NRZ-58292	OXA-48+CTXM-14+TEM-1	234	Germany
GCA_038051685.1	P4; PM-OXA-04	OXA-48+CTXM-14+TEM-1	234	Germany
GCA_046311575.1	Carb-63; NRZ-75157	OXA-48+CTXM-14+VIM-1+TEM-2	269	Germany
GCA_022110455	GBR1; 103223	OXA-48+CTXM-14+TEM-1	269	GreatBritain
GCA_031107755	WI1; 2023GN-00406	OXA-48+CTXM-14+TEM-1	269	USA
GCA_023242195.1	HURS-186083	OXA-48+CTXM-15	298	Spain
GCA_023242175	ESP1; HURS-181823	OXA-48+CTXM-15	298	Spain
GCA_025880885	NOR1	OXA-48	312	Norway
GCA_038070585.1	P5; PM-OXA-05	OXA-48	343	Germany
GCA_030335755.1	Carb-32; NRZ-65277	OXA-48	343	Germany
GCA_046311515.1	Carb-82; NRZ-83375	OXA-48+VEB-6	446	Germany
GCA_039566975.1	P7; PM-OXA-07	OXA-48	461	Germany
GCA_030292485.1	Carb-35	OXA-48	461	Germany
GCA_030336205.1	Carb-11; NRZ-30483	OXA-48+CTXM-14	479	Germany
GCA_030335745.1	Carb-34	OXA-48+CTXM-14	479	Germany
GCF_039566995.1	P1; PM-OXA-01	OXA-48+CTXM-14	479	Germany
GCA_039566985.1	P6; PM-OXA-06	OXA-48+CTXM-14	479	Germany
GCA_038070595.1	P2; PM-OXA-02	OXA-48+TEM-1	484	Germany
GCA_030336085.1	Carb-14; NRZ-33558	OXA-48+TEM-1	484	Germany

Table S6. *In silico* prediction of genomic islands (GIs) in strain Pm GR-1.

Genomic islands (GIs)	Size (bp)	Start position	End position	Locus Tag (Gene)
PGI-30; cluster-1	16,055	1,812	17,867	LIMFOD_09010,LIMFOD_09005,LIMFOD_09000(sul1),LIMFOD_08995(qacEdelta1),LIMFOD_08990(aadA5),LIMFOD_08985(dfrA17),LIMFOD_08980(intI1),LIMFOD_08975(eAL),LIMFOD_08970(tnp),LIMFOD_08965,LIMFOD_08960,LIMFOD_08955(merD),LIMFOD_08950(merA,LIMFOD_08945,LIMFOD_08940(merP),LIMFOD_08935(merR),LIMFOD_08930,LIMFOD_08925,LIMFOD_08920(tnp),LIMFOD_08915
GI-2; cluster-8	23,151	86,292	109,443	LIMFOD_08600(atpI),LIMFOD_08595(atpB),LIMFOD_08590(atpE).LIMFOD_08585(atpF),LIMFOD_08580(atpH),LIMFOD_08575(atpA),LIMFOD_08570(atpG),LIMFOD_08565(atpD),LIMFOD_08560(atpC),LIMFOD_08555(glmU),LIMFOD_08550(glmS),LIMFOD_08545(tnsA),LIMFOD_08540(tnsB),LIMFOD_08535(tnsC)LIMFOD_08530(tnsD),LIMFOD_08525(tnsE),LIMFOD_08520,LIMFOD_08515(ybfA),LIMFOD_08510,LIMFOD_08505(aadA1),LIMFOD_08500(sat2),LIMFOD_08495(dfrA1)LIMFOD_08490(intI2)
cluster-9	20,499	193,447	213,946	LIMFOD_08150,LIMFOD_08145,LIMFOD_08140(phd),LIMFOD_08135,LIMFOD_08130,LIMFOD_08125,LIMFOD_08120,LIMFOD_08115,LIMFOD_08110,LIMFOD_08105,LIMFOD_08100,LIMFOD_08095,LIMFOD_08090,LIMFOD_08085,LIMFOD_08080,LIMFOD_08075,LIMFOD_08070,LIMFOD_08065,LIMFOD_08060(alpA,LIMFOD_08055,LIMFOD_08050
GI-3; cluster-10	38,214	250,665	288,879	LIMFOD_07860(trmL),LIMFOD_07855(ugd),LIMFOD_07850,LIMFOD_07845,LIMFOD_07840,LIMFOD_07835,LIMFOD_07830,LIMFOD_07825,LIMFOD_07820,LIMFOD_07815,LIMFOD_07810,LIMFOD_07805,LIMFOD_07800,LIMFOD_07795(vioA),LIMFOD_07790(cpxA),LIMFOD_07785(cpxR),LIMFOD_07780(cpxP),LIMFOD_07775,LIMFOD_07770,LIMFOD_07765,LIMFOD_07760,LIMFOD_07755,LIMFOD_07750,LIMFOD_07745,LIMFOD_07740,LIMFOD_07735(dksA),LIMFOD_07730,LIMFOD_07725,LIMFOD_07720,LIMFOD_07715,LIMFOD_07710,LIMFOD_07705,LIMFOD_07700,LIMFOD_07695,LIMFOD_07690(yjcR,LIMFOD_07685,LIMFOD_07680,LIMFOD_07675,LIMFOD_07670,LIMFOD_07665(gpX),LIMFOD_07660,LIMFOD_07655,LIMFOD_07650,LIMFOD_07645,LIMFOD_07640,LIMFOD_07635(gpV),LIMFOD_07630,LIMFOD_07625,LIMFOD_07620(gpI)
GI-4; cluster-2	66,718	1,126,900	1,193,618	LIMFOD_03705(tolC),LIMFOD_03700(emrA),LIMFOD_03695(arpD),LIMFOD_03690(zapA),LIMFOD_03685,LIMFOD_03680,LIMFOD_03675,LIMFOD_03670(hipB),LIMFOD_03665(argE),LIMFOD_03660,LIMFOD_03655(pgmB),LIMFOD_03650(aTH1),LIMFOD_03645(treB),LIMFOD_03640(treR),LIMFOD_03635,LIMFOD_03630(araC),LIMFOD_03625,LIMFOD_03620,LIMFOD_03615,LIMFOD_03610,LIMFOD_03605,LIMFOD_03600,LIMFOD_03595(potE,LIMFOD_03590(speF,LIMFOD_03585,LIMFOD_03580,LIMFOD_03575,LIMFOD_03570,LIMFOD_03565(mcrA),LIMFOD_03560,LIMFOD_03555,LIMFOD_03550,LIMFOD_03545,LIMFOD_03540,LIMFOD_03535,LIMFOD_03530,LIMFOD_03525,LIMFOD_03520(tra5),LIMFOD_03515(trnF),LIMFOD_03510(parA),LIMFOD_03505,LIMFOD_03500(dnaB-PI),LIMFOD_03495(parB),LIMFOD_03490,LIMFOD_03485,LIMFOD_03480,LIMFOD_03475(dfsB),LIMFOD_03470,LIMFOD_03465,LIMFOD_03460(topB)
GI-5; PGI-31; cluster-3	37,603	1,233,058	1,270,661	LIMFOD_03325,LIMFOD_03320,LIMFOD_03315(cirA),LIMFOD_03310(tnpA),LIMFOD_03305,LIMFOD_03300,LIMFOD_03295,LIMFOD_03290,LIMFOD_03285,LIMFOD_03280,LIMFOD_03275(traD),LIMFOD_03270,LIMFOD_03265,LIMFOD_03260,LIMFOD_03255,LIMFOD_03250,LIMFOD_03245,LIMFOD_03240,LIMFOD_03235,LIMFOD_03230,LIMFOD_03225,LIMFOD_03220,LIMFOD_03215(virB4),LIMFOD_03210(taaP),LIMFOD_03205,LIMFOD_03200,LIMFOD_0319,LIMFOD_03190,LIMFOD_03185(traG),LIMFOD_03180,LIMFOD_03175,LIMFOD_03170,LIMFOD_03165(STAXI),LIMFOD_03160(EFASI),LIMFOD_03155,LIMFOD_03150,LIMFOD_03145(STAXI,LIMFOD_03140,LIMFOD_03135,LIMFOD_03130(hrpB1),LIMFOD_03125,LIMFOD_03120,LIMFOD_03115(ardC),LIMFOD_03110(ypjF),LIMFOD_03105
GI-7; cluster-17	10,598	1,578,197	1,588,795	LIMFOD_01565,LIMFOD_01560,LIMFOD_01555,LIMFOD_01550,LIMFOD_01545(ymfN),LIMFOD_01540(gene=beeE),LIMFOD_01535,LIMFOD_01530,LIMFOD_01525,LIMFOD_01520,LIMFOD_01515,LIMFOD_01510,LIMFOD_01505,LIMFOD_01500,LIMFOD_01495,LIMFOD_01490
GI-8; cluster-11	22,369	1,719,536	1,741,905	LIMFOD_00945(hcp),LIMFOD_00940;(vgrG),LIMFOD_00935,LIMFOD_00930,LIMFOD_00925,LIMFOD_00920,LIMFOD_00915,LIMFOD_00910,LIMFOD_00905,LIMFOD_00900,LIMFOD_00895,LIMFOD_00890,LIMFOD_00885,LIMFOD_00880,LIMFOD_00875(tetR),LIMFOD_00870(istA),LIMFOD_00865,LIMFOD_00860,LIMFOD_00855,LIMFOD_00850,LIMFOD_00845(hicB),LIMFOD_00840,LIMFOD_00835,LIMFOD_00830
cluster-4	9,144	1,790,545	1,799,689	LIMFOD_00600(ubiE),LIMFOD_00595,LIMFOD_00590(ryhB),LIMFOD_00585,LIMFOD_00580,LIMFOD_00575(cfa),LIMFOD_00570,LIMFOD_00565(ydeN),LIMFOD_00560(lysE),LIMFOD_00555,LIMFOD_00550(hicB),LIMFOD_00545,LIMFOD_00540,LIMFOD_00535,LIMFOD_00530,LIMFOD_00525
GI-9; cluster-5	18,853	1,879,515	1,898,368	LIMFOD_00170,LIMFOD_00165,LIMFOD_00160(tnp),LIMFOD_00155,LIMFOD_00150,LIMFOD_00145,LIMFOD_00140,LIMFOD_00135,LIMFOD_00130(lexA),LIMFOD_00125,LIMFOD_00120,LIMFOD_00115(xRE),LIMFOD_00110(hipB),LIMFOD_00105(sfsB),LIMFOD_00100(marR),LIMFOD_00095(yhdJ),LIMFOD_00090,LIMFOD_00085,LIMFOD_00080,LIMFOD_00075,LIMFOD_00070,LIMFOD_00065,LIMFOD_00060,LIMFOD_00055,LIMFOD_00050,LIMFOD_00045,LIMFOD_00040
GI-11; cluster-18	16,866	2,179,758	2,196,624	LIMFOD_20210,LIMFOD_20215,LIMFOD_20220,LIMFOD_20225,LIMFOD_20230,LIMFOD_20235(fliS),LIMFOD_20240,LIMFOD_20245(glgD),LIMFOD_20250,LIMFOD_20255,LIMFOD_20260,LIMFOD_20265,LIMFOD_20270,LIMFOD_20275,LIMFOD_20280,LIMFOD_20285,LIMFOD_20290,LIMFOD_20295,LIMFOD_20300,LIMFOD_20305,LIMFOD_20310,LIMFOD_20315,LIMFOD_20320,LIM

				FOD_20325,LIMFOD_20330
GI-13; PGI-32; cluster-6	15,296	2,212,846	2,228,142	LIMFOD_09135,LIMFOD_09140,LIMFOD_09145,LIMFOD_09150,LIMFOD_09155,LIMFOD_09160,LIMFOD_09165,LIMFOD_09170,LIMFOD_09175,LIMFOD_09180,LIMFOD_09185,LIMFOD_09190,LIMFOD_09195,LIMFOD_09200,LIMFOD_09205,LIMFOD_09210,LIMFOD_09215,LIMFOD_09220(recT),LIMFOD_09225,LIMFOD_09230,LIMFOD_09235(ssb),LIMFOD_09240(xkdX),LIMFOD_09245,LIMFOD_09250,LIMFOD_09255,LIMFOD_09260,LIMFOD_09265,LIMFOD_09270,LIMFOD_09275,LIMFOD_09280,LIMFOD_09285
GI-14; cluster-12	12,910	2,479,205	2,492,115	LIMFOD_10410(rpsU),LIMFOD_10415(dnaG),LIMFOD_10420(rpoD),LIMFOD_10425(trnI),LIMFOD_10430,LIMFOD_10435,LIMFOD_10440,LIMFOD_10445,LIMFOD_10450,LIMFOD_10455,LIMFOD_10460,LIMFOD_10465,LIMFOD_10470(yneE)
cluster-13	11,906	2,610,252	2,622,158	LIMFOD_11015(ygcG),LIMFOD_11020(lemA),LIMFOD_11025(ubiH),LIMFOD_11030(yqiK),LIMFOD_11035,LIMFOD_11040(feaR)
cluster-7	21,514	2,788,685	2,810,199	LIMFOD_11805(thiH),LIMFOD_11810(rpnC),LIMFOD_11815(rpoC),LIMFOD_11820(rpoB),LIMFOD_11825(P26),LIMFOD_11830(rplL),LIMFOD_11835(rplJ),LIMFOD_11840(rplA),LIMFOD_11845(rplK),LIMFOD_11850(nusG),LIMFOD_11855(secE),LIMFOD_11860(tuf),LIMFOD_11865(fusA),LIMFOD_11870(rpsG),LIMFOD_11875(rpsL),LIMFOD_11880(tusB),LIMFOD_11885(tusC),LIMFOD_11890(tusD)
PGI-33; cluster-14	65,360	3,018,667	3,084,027	LIMFOD_12865,LIMFOD_12870(idsD),LIMFOD_12875(idsE),LIMFOD_12880(pAAR),LIMFOD_12885,LIMFOD_12890,LIMFOD_12895,LIMFOD_12900,LIMFOD_12905(ecpB),LIMFOD_12910(ecpA),LIMFOD_12915(hipB),LIMFOD_12920(rhuM),LIMFOD_12925,LIMFOD_12930,LIMFOD_12935(yeeT),LIMFOD_12940(radC),LIMFOD_12945,LIMFOD_12950,LIMFOD_12955,LIMFOD_12960,LIMFOD_12965(yeeP),LIMFOD_12970,LIMFOD_12975(tnp),LIMFOD_12980(tnp),LIMFOD_12985,LIMFOD_12990(alpA,LIMFOD_12995,LIMFOD_13000,LIMFOD_13005,LIMFOD_13010(tnp),LIMFOD_13015(tnpB),LIMFOD_13020,LIMFOD_13025(tnp),LIMFOD_13030(tnp),LIMFOD_13035(catA1),LIMFOD_13040,LIMFOD_13045,LIMFOD_13050,LIMFOD_13055,LIMFOD_13060(tnp),LIMFOD_13065,LIMFOD_13070(blaTEM-1),LIMFOD_13075(tnp),LIMFOD_13080[aph(3')Ia],LIMFOD_13085(tnp),LIMFOD_13090(eAL),LIMFOD_13095(intI1),LIMFOD_13100(dfrA17),LIMFOD_13105(aadA5),LIMFOD_13110(qacEdelta1,LIMFOD_13115(sul1),LIMFOD_13120,LIMFOD_13125,LIMFOD_13130(tniA),LIMFOD_13135,LIMFOD_13140(merD),LIMFOD_13145(merA),LIMFOD_13150,LIMFOD_13155(merP),LIMFOD_13160(merR),LIMFOD_13165,LIMFOD_13170(RNA-OUT),LIMFOD175(blaOXA-48),LIMFOD_13185(RNA-OUT),LIMFOD_13190(tnp)
GI-34; cluster-1	31,265	3,086,222	3,117,487	LIMFOD_13220(umuC),LIMFOD_13225(copG),LIMFOD_13230(tgtA5),LIMFOD_13235,LIMFOD_13240(relB),LIMFOD_13245,LIMFOD_13250,LIMFOD_13255,LIMFOD_13260,LIMFOD_13265,LIMFOD_13270(mrr),LIMFOD_13275,LIMFOD_13280,LIMFOD_13285,LIMFOD_13290(parA),LIMFOD_13295,LIMFOD_13300,LIMFOD_13305,LIMFOD_13310,LIMFOD_13315,LIMFOD_13320(radC),LIMFOD_13325(korC),LIMFOD_13330(cbiX),LIMFOD_13335,LIMFOD_13340(blaCTX-M-14),LIMFOD_13345(tnp),LIMFOD_13350,LIMFOD_13355(rmoA),LIMFOD_13360,LIMFOD_13365(tnp),LIMFOD_13370[(aph(3'')-Ib],LIMFOD_13375(aph(6)-Id,LIMFOD_13380(tnp),LIMFOD_13385(RNA-OUT),LIMFOD_13390(tnp),LIMFOD_13395(RNA-OUT),LIMFOD_13400[aac(3)-IId],LIMFOD_13405,LIMFOD_13410(trnT),LIMFOD_13415,LIMFOD_13420(tnp
GI-18; cluster-5	22,521	3,941,390	3,963,911	LIMFOD_17405,LIMFOD_17410,LIMFOD_17415,LIMFOD_17420,LIMFOD_17425,LIMFOD_17430,LIMFOD_17435,LIMFOD_17440,LIMFOD_17445,LIMFOD_17450,LIMFOD_17455,LIMFOD_17460,LIMFOD_17465,LIMFOD_17470,LIMFOD_17475,LIMFOD_17480,LIMFOD_17485,LIMFOD_17490,LIMFOD_17495,LIMFOD_17500,LIMFOD_17505,LIMFOD_17510,LIMFOD_17515,LIMFOD_17520,LIMFOD_17525,LIMFOD_17530(icd),LIMFOD_17535
GI-19; cluster-15	8,769	4,195,653	4,204,422	LIMFOD_18645,LIMFOD_18650,LIMFOD_18655,LIMFOD_18660(dAP2),LIMFOD_18665,LIMFOD_18670,LIMFOD_18675(yfdV),LIMFOD_18680
GI-20; PGI-35; cluster-6	32,709	4,229,464	4,262,173	LIMFOD_18785,LIMFOD_18790,LIMFOD_18795,LIMFOD_18800,LIMFOD_18805,LIMFOD_18810,LIMFOD_18815(xkdX),LIMFOD_18820(ssb),LIMFOD_18825,LIMFOD_18830,LIMFOD_18835(recT),LIMFOD_18840,LIMFOD_18845,LIMFOD_18850,LIMFOD_18855,LIMFOD_18860,LIMFOD_18865,LIMFOD_18870,LIMFOD_18875,LIMFOD_18880,LIMFOD_18885,LIMFOD_18890,LIMFOD_18895,LIMFOD_18900,LIMFOD_18905,LIMFOD_18910,LIMFOD_18915,LIMFOD_18920(marR),LIMFOD_18925,LIMFOD_18930(ninB),LIMFOD_18935,LIMFOD_18940,LIMFOD_18945,LIMFOD_18950,LIMFOD_18955,LIMFOD_18960,LIMFOD_18965,LIMFOD_18970,LIMFOD_18975,LIMFOD_18980,LIMFOD_18985,LIMFOD_18990,LIMFOD_20380,LIMFOD_20385,LIMFOD_20390,LIMFOD_20395,LIMFOD_20400,LIMFOD_20405(xkdX),LIMFOD_20410,LIMFOD_20415,LIMFOD_20420,LIMFOD_20425(recT),LIMFOD_20430,LIMFOD_20435,LIMFOD_20440,LIMFOD_20445,LIMFOD_20450,LIMFOD_20455,LIMFOD_20460,LIMFOD_20465,LIMFOD_20470,LIMFOD_20475,LIMFOD_20480,LIMFOD_20485,LIMFOD_20490,LIMFOD_20495,LIMFOD_20500
GI-21; cluster-19	49,465	4,265,485	4,314,950	LIMFOD_20560,LIMFOD_20565,LIMFOD_20570,LIMFOD_20575,LIMFOD_20580,LIMFOD_20585,LIMFOD_20590,LIMFOD_20595,LIMFOD_20600,LIMFOD_20605,LIMFOD_20610,LIMFOD_20615,LIMFOD_20620,LIMFOD_20625,LIMFOD_20630,LIMFOD_20635,LIMFOD_20640,LIMFOD_20645,LIMFOD_20650,LIMFOD_20655,LIMFOD_20660,LIMFOD_20665,LIMFOD_20670,LIMFOD_20675,LIMFOD_20680,LIMFOD_20685,LIMFOD_20690,LIMFOD_20695,LIMFOD_20700,LIMFOD_20705,LIMFOD_20710,LIMFOD_20715,LIMFOD_20720,LIMFOD_20725,LIMFOD_20730,LIMFOD_20735,LIMFOD_20740,LIMFOD_20745,LIMFOD_20750,LIMFOD_20755(tnpR),LIMFOD_20760(blaTEM2),LIMFOD_20765(sok),LIMFOD_20770,LIMFOD_20775,LIMFOD_20780,LIMFOD_20785(hns),LIMFOD_20790(topB),LIMFOD_20795(pcfJ),LIMFOD_20800,LIMFOD_20805,LIMFOD_20810,LIMFOD_

				20815(trbM),LIMFOD_20820,LIMFOD_20825(traG),LIMFOD_20830(virB11),LIMFOD_20835,LIMFOD_20840(virB9),LIMFOD_20845(virB8),LIMFOD_20850,LIMFOD_20855
GI-22; cluster-20	8,759	4,322,672	4,331,431	LIMFOD_20910(relE),LIMFOD_20915(cotH),LIMFOD_20920,LIMFOD_20925,LIMFOD_20930,LIMFOD_20935,LIMFOD_20940,LIMFOD_20945,LIMFOD_20950,LIMFOD_20955,LIMFOD_20960,LIMFOD_20965(dnaJ),LIMFOD_20970,LIMFOD_20975,LIMFOD_20980,LIMFOD_20985(scpB),LIMFOD_20990,LIMFOD_20995(copG),LIMFOD_20910(relE),LIMFOD_20915(cotH),LIMFOD_20920,LIMFOD_20925,LIMFOD_20930,LIMFOD_20935,LIMFOD_20940,LIMFOD_20945,LIMFOD_20950,LIMFOD_20955,LIMFOD_20960,LIMFOD_20965(dnaJ),LIMFOD_20970,LIMFOD_20975,LIMFOD_20980,LIMFOD_20985(scpB),LIMFOD_20990,LIMFOD_20995(copG)

Table S7. *In silico* predictions of ARGs among the eight studied ST135 *P. miralis* isolates.

AMR drug class	AMR genes	GR-1	P3	NRZ-36257	AHEPA-923	P8	Carb-25	Carb-89	NY-1
β-lactams	*bla*$_{CTX-M-14}$	■	■	■					
	bla$_{CTX-M-15}$							■	■
	bla$_{CTX-M-65}$					■	■		
	bla$_{NDM-1}$							■	
	bla$_{OXA-1}$					■	■		■
	bla$_{OXA-48}$	■	■	■		■	■	■	■
	bla$_{OXA-9}$							■	
	bla$_{TEM-1A}$							■	
	bla$_{TEM-1B}$		■			■	■		■
	bla$_{TEM-2}$	■	■	■	■				
	bla$_{VEB-6}$							■	
	bla$_{VIM-78}$				■				
	ble								■
Aminoglycosides	*aac(3)-IIa*								■
	aac(3)-IId	■	■	■	■	■	■		
	aac(3)-IVa				■	■	■		
	aac(6')-IIc				■				
	aac(6')-Ib							■	
	aadA14				■				
	aadA2		■	■					
	aadA5	■	■	■	■	■	■	■	■
	ant(3'')-Ia	■	■	■	■	■	■	■	■
	aph(3'')-Ib_2	■	■						
	aph(3'')-Ib_5		■	■	■	■	■		
	aph(3')-Ia_1	■				■	■		■
	aph(3')-Ia_7				■				
	aph(3')-VI							■	
	aph(3')-VIb		■	■					
	aph(4)-Ia					■	■		
	aph(6)-Id	■	■	■	■	■	■		
	armA							■	
Aminoglycosides/ Quinolones	*aac(6')-Ib-cr*					■	■		■
Rifamycin	*ARR-3_4*					■	■		
Phenicols	*catA1*	■	■	■	■	■	■	■	■
	catB3					■	■		
	cat	■	■	■	■	■	■	■	■
	floR					■	■		
Trimethoprim	*dfrA12*		■	■					
	dfrA17	■	■	■	■	■	■	■	■
	dfrA1	■	■	■	■	■	■	■	■
Fosfomycin	*fosA3*					■	■		
Lincosamides	*lnu(F)*				■				
Macrolides	*mph(A)*		■	■					■
	mph(E)							■	
Macrolides/ Streptogramines	*msr(E)*							■	
Quinolone	*qnrA1*							■	
	qnrS1							■	
Streptothricin	*sat2*	■	■	■	■	■	■	■	■
Sulfonamides	*sul1*	■	■	■	■	■	■	■	■
	sul2_2		■	■	■	■		■	
	sul2_3		■	■		■			
Tetracycline	*tet(A)*							■	
	tet(J)	■	■	■	■	■	■	■	■

Table S8. *In silico* predictions of stress/heavy metal resistance genes among the eight studied ST135 *P. miralis* isolates.

Stress Resistance Class	Stress Resistance genes	GR-1	P3	NRZ_36257	AHEPA 923	P8	Carb-25	Carb-89	NY-1
Mercury	*merA*	■	■		■	■	■	■	
	merC	■	■	■	■	■	■	■	■
	merD	■	■	■	■	■	■	■	■
	merE	■	■	■	■	■	■	■	■
	merP	■	■	■	■	■	■	■	■
	merR	■	■	■	■	■	■	■	■
	merT	■	■	■	■	■	■	■	■
Tellurium	*terD*	■	■	■	■	■	■	■	■
	terZ	■	■	■	■	■	■	■	■
Arsenic	*arsA*								■
	arsB								■
	arsC								■
	arsD								■
	arsR								■
Copper	*pcoA*								■
	pcoB								■
	pcoC								■
	pcoD								■
	pcoE								■
	pcoR								■
	pcoS								■
Copper/ Silver	*silA*								■
	silB								■
	silE								■
	silF								■
	silP								■
	silR								■
	silS								■
	silP								■
Heat shock/ survival proteins	*clpK*								■
	hsp20								■

Table S9. BLASTp results of the predicted proteins of strain Pm GR-1 genome and the VFDB dataset. The proteins showing >60% similarity are shown.

Query accession dot version	Subject accession	Percentage of identical matches	All subject title(s),
LIMFOD_00945	VFG038365(gb| WP_011705044)	89.535	(hcp1) HcpA-like protein [T6SS (VF0480) - Effector delivery system (VFC0086)] [Aeromonas hydrophila subsp. hydrophila ATCC 7966]
LIMFOD_18260	VFG038365(gb| WP_011705044)	89.535	(hcp1) HcpA-like protein [T6SS (VF0480) - Effector delivery system (VFC0086)] [Aeromonas hydrophila subsp. hydrophila ATCC 7966]
LIMFOD_13975	VFG007136(gb| WP_001142947)	89.474	(hcp-1) type VI secretion system effector Hcp-2 [VAS T6SS (VF0335) - Effector delivery system (VFC0086)] [Vibrio cholerae O1 biovar El Tor str. N16961]
LIMFOD_00945	VFG038369(gb| WP_011705706)	88.953	(hcp) hemolysin co-regulated protein [T6SS (VF0480) - Effector delivery system (VFC0086)] [Aeromonas hydrophila subsp. hydrophila ATCC 7966]
LIMFOD_18260	VFG038369(gb| WP_011705706)	88.953	(hcp) hemolysin co-regulated protein [T6SS (VF0480) - Effector delivery system (VFC0086)] [Aeromonas hydrophila subsp. hydrophila ATCC 7966]
LIMFOD_16295	VFG049018(gb| WP_002913007)	88.889	(rcsB) transcriptional regulator RcsB [RcsAB (VF0571) - Regulation (VFC0301)] [Klebsiella pneumoniae subsp. pneumoniae NTUH-K2044]
LIMFOD_12850	VFG038365(gb| WP_011705044)	88.372	(hcp1) HcpA-like protein [T6SS (VF0480) - Effector delivery system (VFC0086)] [Aeromonas hydrophila subsp. hydrophila ATCC 7966]
LIMFOD_12850	VFG038369(gb| WP_011705706)	87.791	(hcp) hemolysin co-regulated protein [T6SS (VF0480) - Effector delivery system (VFC0086)] [Aeromonas hydrophila subsp. hydrophila ATCC 7966]
LIMFOD_02305	VFG000478(gb| NP_459678)	86.395	(fur) ferric iron uptake transcriptional regulator [Fur (VF0113) - Regulation (VFC0301)] [Salmonella enterica subsp. enterica serovar Typhimurium str. LT2]
LIMFOD_01725	VFG048830(gb| WP_014907233)	85.928	(gndA) NADP-dependent phosphogluconate dehydrogenase [Capsule (VF0560) - Immune modulation (VFC0258)] [Klebsiella pneumoniae subsp. pneumoniae NTUH-K2044]
LIMFOD_15850	VFG002346(gb| WP_005160438)	84.231	(flgG) flagellar basal-body rod protein FlgG [Flagella (VF0394) - Motility (VFC0204)] [Yersinia enterocolitica subsp. enterocolitica 8081]
LIMFOD_15915	VFG043206(gb| WP_005164479)	83.721	(cheY) chemotaxis regulatory protein CheY [Flagella (VF0394) - Motility (VFC0204)] [Yersinia enterocolitica subsp. enterocolitica 8081]
LIMFOD_02820	VFG010906(gb| WP_011213314)	83.636	(csrA) carbon storage regulator CsrA [CsrA (VF0261) - Regulation (VFC0301)] [Legionella pneumophila subsp. pneumophila str. Philadelphia 1]
LIMFOD_15695	VFG002319(gb| WP_005160341)	83.544	(fliA) flagellar biosynthesis sigma factor [Flagella (VF0394) - Motility (VFC0204)] [Yersinia enterocolitica subsp. enterocolitica 8081]
LIMFOD_15960	VFG002358(gb| WP_005164496)	83.333	(flhC) flagellar biosynthesis transcription activator FlhC [Flagella (VF0394) - Motility (VFC0204)] [Yersinia enterocolitica subsp. enterocolitica 8081]
LIMFOD_00945	VFG049915(gb| WP_001142968)	83.140	(hcp2/tssD2) Type VI secretion system protein, Hcp family [T6SS (VF0579) - Effector delivery system (VFC0086)] [Shigella sonnei Ss046]
LIMFOD_18260	VFG049915(gb| WP_001142968)	83.140	(hcp2/tssD2) Type VI secretion system protein, Hcp family [T6SS (VF0579) - Effector delivery system (VFC0086)] [Shigella sonnei Ss046]
LIMFOD_12850	VFG049915(gb| WP_001142968)	81.977	(hcp2/tssD2) Type VI secretion system protein, Hcp family [T6SS (VF0579) - Effector delivery system (VFC0086)] [Shigella sonnei Ss046]
LIMFOD_15760	VFG002329(gb| WP_005160381)	81.707	(fliG) flagellar motor switch protein G [Flagella (VF0394) - Motility (VFC0204)] [Yersinia enterocolitica subsp. enterocolitica 8081]
LIMFOD_15940	VFG043210(gb| WP_011816618)	81.046	(cheW) purine-binding chemotaxis protein CheW [Flagella (VF0394) - Motility (VFC0204)] [Yersinia enterocolitica subsp. enterocolitica 8081]
LIMFOD_15965	VFG002359(gb| WP_005164498)	81.034	(flhD) flagellar transcriptional activator FlhD [Flagella (VF0394) - Motility (VFC0204)] [Yersinia enterocolitica subsp. enterocolitica 8081]
LIMFOD_07240	VFG046465(gb| WP_003028672)	80.916	(tufA) elongation factor Tu [EF-Tu (VF0460) - Adherence (VFC0001)] [Francisella tularensis subsp. tularensis SCHU S4]
LIMFOD_11860	VFG046465(gb| WP_003028672)	80.916	(tufA) elongation factor Tu [EF-Tu (VF0460) - Adherence (VFC0001)] [Francisella tularensis subsp. tularensis SCHU S4]
LIMFOD_18115	VFG013465(gb| WP_005693586)	80.212	(kdsA) 2-dehydro-3-deoxyphosphooctonate aldolase [LOS (VF0044) - Immune modulation (VFC0258)] [Haemophilus influenzae Rd KW20]
LIMFOD_15805	VFG002338(gb| WP_042661456)	80.176	(fliP) flagellar biosynthetic protein FliP [Flagella (VF0394) - Motility (VFC0204)] [Yersinia enterocolitica subsp. enterocolitica 8081]
LIMFOD_09705	VFG000477(gb| NP_461845)	79.939	(rpoS) RNA polymerase sigma factor RpoS [RpoS (VF0112) - Regulation (VFC0301)] [Salmonella enterica subsp. enterica serovar Typhimurium str. LT2]
LIMFOD_15770	VFG002331(gb| WP_026018001)	79.646	(fliI) flagellum-specific ATP synthase FliI [Flagella (VF0394) - Motility (VFC0204)] [Yersinia enterocolitica subsp. enterocolitica 8081]
LIMFOD_07855	VFG048797(gb| WP_004175261)	78.866	(ugd) UDP-glucose 6-dehydrogenase [Capsule (VF0560) - Immune modulation (VFC0258)] [Klebsiella pneumoniae subsp. pneumoniae NTUH-K2044]
LIMFOD_15790	VFG002335(gb| WP_005160396)	78.717	(fliM) flagellar motor switch protein FliM [Flagella (VF0394) - Motility (VFC0204)] [Yersinia enterocolitica subsp. enterocolitica 8081]
LIMFOD_00945	VFG007136(gb| WP_001142947)	78.488	(hcp-1) type VI secretion system effector Hcp-2 [VAS T6SS (VF0335) - Effector delivery system (VFC0086)] [Vibrio cholerae O1 biovar El Tor str. N16961]
LIMFOD_18260	VFG007136(gb| WP_001142947)	78.488	(hcp-1) type VI secretion system effector Hcp-2 [VAS T6SS (VF0335) - Effector delivery system (VFC0086)] [Vibrio cholerae O1 biovar El Tor str. N16961]
LIMFOD_02955	VFG013418(gb|	78.125	(gmhA/lpcA) phosphoheptose isomerase [LOS (VF0044) - Immune modulation

	WP_005694260)		(VFC0258)] [Haemophilus influenzae Rd KW20]
LIMFOD_12850	VFG007136(gb| WP_001142947)	77.907	(hcp-1) type VI secretion system effector Hcp-2 [VAS T6SS (VF0335) - Effector delivery system (VFC0086)] [Vibrio cholerae O1 biovar El Tor str. N16961]
LIMFOD_15900	VFG002356(gb| WP_011816614)	77.746	(flhA) flagellar biosynthesis protein FlhA [Flagella (VF0394) - Motility (VFC0204)] [Yersinia enterocolitica subsp. enterocolitica 8081]
LIMFOD_07915	VFG000332(gb| WP_005632797)	77.742	(rfaD) ADP-L-glycero-D-mannoheptose-6-epimerase [LOS (VF0044) - Immune modulation (VFC0258)] [Haemophilus influenzae Rd KW20]
LIMFOD_15870	VFG002350(gb| WP_005168794)	77.612	(flgC) flagellar basal-body rod protein FlgC [Flagella (VF0394) - Motility (VFC0204)] [Yersinia enterocolitica subsp. enterocolitica 8081]
LIMFOD_15810	VFG002339(gb| WP_005160414)	77.528	(fliQ) flagellar biosynthetic protein FliQ [Flagella (VF0394) - Motility (VFC0204)] [Yersinia enterocolitica subsp. enterocolitica 8081]
LIMFOD_00330	VFG038840(gb| WP_005300916)	77.459	(flmH) short chain dehydrogenase/reductase family oxidoreductase [Polar flagella (VF0473) - Motility (VFC0204)] [Aeromonas hydrophila ML09-119]
LIMFOD_15700	VFG002321(gb| WP_011816589)	77.273	(fliC) flagellin [Flagella (VF0394) - Motility (VFC0204)] [Yersinia enterocolitica subsp. enterocolitica 8081]
LIMFOD_00955	VFG038380(gb| WP_043163044)	76.220	(vipB) Type VI secretion system contractile sheath large subunit TssC/VipB [T6SS (VF0480) - Effector delivery system (VFC0086)] [Aeromonas hydrophila subsp. hydrophila ATCC 7966]
LIMFOD_15920	VFG043207(gb| WP_005164481)	76.000	(cheB) chemotaxis-specific methylesterase CheB [Flagella (VF0394) - Motility (VFC0204)] [Yersinia enterocolitica subsp. enterocolitica 8081]
LIMFOD_00955	VFG002093(gb| WP_000108140)	75.891	(vipB/mglB) type VI secretion system tubule-forming protein VipB [VAS T6SS (VF0335) - Effector delivery system (VFC0086)] [Vibrio cholerae O1 biovar El Tor str. N16961]
LIMFOD_04365	VFG049144(gb| WP_002892069)	75.475	(acrB) acriflavine resistance protein B [AcrAB (VF0568) - Antimicrobial activity/Competitive advantage (VFC0325)] [Klebsiella pneumoniae subsp. pneumoniae NTUH-K2044]
LIMFOD_15845	VFG002345(gb| WP_005160434)	75.431	(flgH) flagellar L-ring protein precursor FlgH [Flagella (VF0394) - Motility (VFC0204)] [Yersinia enterocolitica subsp. enterocolitica 8081]
LIMFOD_15915	VFG002525(gb| WP_004185006)	75.000	(cheY) chemotaxis protein CheY [Flagella (VF0430) - Motility (VFC0204)] [Burkholderia pseudomallei K96243]
LIMFOD_19495	VFG013412(gb| WP_005693459)	75.000	(lpxC) UDP-3-O-(R-3-hydroxymyristoyl)-N-acetylglucosamine deacetylase [LOS (VF0044) - Immune modulation (VFC0258)] [Haemophilus influenzae Rd KW20]
LIMFOD_02785	VFG018241(gb| WP_001130227)	74.854	(luxS) S-ribosylhomocysteinase [AI-2 (VF0406) - Biofilm (VFC0271)] [Vibrio cholerae O1 biovar El Tor str. N16961]
LIMFOD_10980	VFG001855(gb| WP_197535493)	74.669	(htpB) Hsp60, 60K heat shock protein HtpB [Hsp60 (VF0159) - Adherence (VFC0001)] [Legionella pneumophila subsp. pneumophila str. Philadelphia 1]
LIMFOD_13975	VFG038365(gb| WP_011705044)	74.194	(hcp1) HcpA-like protein [T6SS (VF0480) - Effector delivery system (VFC0086)] [Aeromonas hydrophila subsp. hydrophila ATCC 7966]
LIMFOD_13975	VFG038369(gb| WP_011705706)	74.194	(hcp) hemolysin co-regulated protein [T6SS (VF0480) - Effector delivery system (VFC0086)] [Aeromonas hydrophila subsp. hydrophila ATCC 7966]
LIMFOD_15910	VFG043205(gb| WP_005164477)	74.057	(cheZ) chemotaxis regulator CheZ [Flagella (VF0394) - Motility (VFC0204)] [Yersinia enterocolitica subsp. enterocolitica 8081]
LIMFOD_09705	VFG043648(gb| NP_252312)	72.563	(rpoS) RNA polymerase sigma factor RpoS [Type IV pili (VF0082) - Adherence (VFC0001)] [Pseudomonas aeruginosa PAO1]
LIMFOD_15840	VFG002344(gb| WP_042661458)	72.118	(flgI) flagellar P-ring protein precursor FlgI [Flagella (VF0394) - Motility (VFC0204)] [Yersinia enterocolitica subsp. enterocolitica 8081]
LIMFOD_01085	VFG038845(gb| WP_016351249)	70.850	(nueA) NeuA protein [Polar flagella (VF0473) - Motility (VFC0204)] [Aeromonas hydrophila ML09-119]
LIMFOD_15955	VFG043213(gb| WP_005164494)	70.588	(motA) flagellar motor protein MotA [Flagella (VF0394) - Motility (VFC0204)] [Yersinia enterocolitica subsp. enterocolitica 8081]
LIMFOD_15795	VFG002336(gb| WP_005160399)	70.504	(fliN) flagellar motor switch protein FliN [Flagella (VF0394) - Motility (VFC0204)] [Yersinia enterocolitica subsp. enterocolitica 8081]
LIMFOD_14785	VFG013346(gb| WP_005693178)	70.345	(galU) glucosephosphate uridylyltransferase [LOS (VF0044) - Immune modulation (VFC0258)] [Haemophilus influenzae Rd KW20]
LIMFOD_13975	VFG049915(gb| WP_001142968)	70.000	(hcp2/tssD2) Type VI secretion system protein, Hcp family [T6SS (VF0579) - Effector delivery system (VFC0086)] [Shigella sonnei Ss046]
LIMFOD_10270	VFG051131(gb| WP_006191504.1)	70.000	(bmaC) autotransporter outer membrane beta-barrel domain-containing protein [BmaC (VF1341) - Adherence (VFC0001)] [Brucella suis 1330]
LIMFOD_19610	VFG038915(gb| WP_011705264)	69.565	(rtxH) conserved hypothetical protein in rtx gene loci [RtxA (VF0482) - Exotoxin (VFC0235)] [Aeromonas hydrophila subsp. hydrophila ATCC 7966]
LIMFOD_00205	VFG000475(gb| NP_460201)	69.507	(phoP) response regulator in two-component regulatory system with PhoQ [PhoPQ (VF0111) - Regulation (VFC0301)] [Salmonella enterica subsp. enterica serovar Typhimurium str. LT2]
LIMFOD_10350	VFG000331(gb| WP_005693548)	69.313	(rfaE) ADP-heptose synthase [LOS (VF0044) - Immune modulation (VFC0258)] [Haemophilus influenzae Rd KW20]
LIMFOD_01180	VFG049124(gb| WP_012737540)	69.253	(allD) ureidoglycolate dehydrogenase [Allantion utilization (VF0572) - Nutritional/Metabolic factor (VFC0272)] [Klebsiella pneumoniae subsp. pneumoniae NTUH-K2044]
LIMFOD_15945	VFG002531(gb| WP_011205291)	68.622	(cheA) chemotaxis two-component sensor kinase CheA [Flagella (VF0430) - Motility (VFC0204)] [Burkholderia pseudomallei K96243]

LIMFOD_00730	VFG001443(gb| AAF37887)	68.508	(ompA) outer membrane protein A [OmpA (VF0236) - Invasion (VFC0083)] [Escherichia coli O18:K1:H7 str. RS218]
LIMFOD_09705	VFG001866(gb| WP_015444568)	68.478	(rpoS) RNA polymerase sigma factor RpoS [RpoS (VF0166) - Regulation (VFC0301)] [Legionella pneumophila subsp. pneumophila str. Philadelphia 1]
LIMFOD_16355	VFG037028(gb| WP_002218620)	68.288	(katA) catalase [KatA (VF0454) - Stress survival (VFC0282)] [Neisseria meningitidis MC58]
LIMFOD_15940	VFG002530(gb| WP_004199265)	67.925	(cheW) chemotaxis protein CheW [Flagella (VF0430) - Motility (VFC0204)] [Burkholderia pseudomallei K96243]
LIMFOD_15770	VFG002499(gb| WP_004197164)	67.699	(fliI) flagellum-specific ATP synthase FliI [Flagella (VF0430) - Motility (VFC0204)] [Burkholderia pseudomallei K96243]
LIMFOD_15850	VFG002513(gb| WP_004197257)	67.308	(flgG) flagellar basal body rod protein FlgG [Flagella (VF0430) - Motility (VFC0204)] [Burkholderia pseudomallei K96243]
LIMFOD_15690	VFG002318(gb| WP_005160338)	67.296	(fliZ) alternative sigma factor regulatory protein [Flagella (VF0394) - Motility (VFC0204)] [Yersinia enterocolitica subsp. enterocolitica 8081]
LIMFOD_01105	VFG013248(gb| WP_010868928)	67.014	(msbA) lipid transporter ATP-binding/permease [LOS (VF0044) - Immune modulation (VFC0258)] [Haemophilus influenzae Rd KW20]
LIMFOD_14315	VFG001867(gb| WP_016357051)	66.492	(sodB) superoxide dismutase [SodB (VF0169) - Stress survival (VFC0282)] [Legionella pneumophila subsp. pneumophila str. Philadelphia 1]
LIMFOD_05150	VFG000274(gb| WP_000238762)	66.327	(ureG) urease accessory protein (ureG) [Urease (VF0050) - Stress survival (VFC0282)] [Helicobacter pylori 26695]
LIMFOD_06865	VFG047039(gb| WP_003018140)	66.207	(wbtL) glucose-1-phosphate thymidylyltransferase [LPS (VF0542) - Immune modulation (VFC0258)] [Francisella tularensis subsp. tularensis SCHU S4]
LIMFOD_15920	VFG002526(gb| WP_004198645)	66.102	(cheB) chemotaxis-specific methylesterase [Flagella (VF0430) - Motility (VFC0204)] [Burkholderia pseudomallei K96243]
LIMFOD_11985	VFG042734(gb| NP_249343)	65.842	(vfr) cAMP-regulatory protein [Type IV pili (VF0082) - Adherence (VFC0001)] [Pseudomonas aeruginosa PAO1]
LIMFOD_08755	VFG046610(gb| WP_003020688)	65.766	(rpe) ribulose-phosphate 3-epimerase [Capsule (VF0543) - Immune modulation (VFC0258)] [Francisella tularensis subsp. tularensis SCHU S4]
LIMFOD_17225	VFG000121(gb| NP_249453)	65.625	(algU) alginate biosynthesis protein AlgZ/FimS [Alginate (VF0091) - Biofilm (VFC0271)] [Pseudomonas aeruginosa PAO1]
LIMFOD_15915	VFG007568(gb| WP_000697869)	65.574	(cheY) chemotaxis protein CheY [Flagella (VF0519) - Motility (VFC0204)] [Vibrio cholerae O1 biovar El Tor str. N16961]
LIMFOD_15805	VFG002494(gb| WP_011204732)	65.455	(fliP) flagellar biosynthesis protein FliP [Flagella (VF0430) - Motility (VFC0204)] [Burkholderia pseudomallei K96243]
LIMFOD_15055	VFG043634(gb| WP_011081076)	65.217	(VV1_RS15610) CpaF family protein [Flp pili (VF0612) - Adherence (VFC0001)] [Vibrio vulnificus CMCP6]
LIMFOD_15945	VFG043211(gb| WP_032902679)	65.199	(cheA) chemotaxis protein CheA [Flagella (VF0394) - Motility (VFC0204)] [Yersinia enterocolitica subsp. enterocolitica 8081]
LIMFOD_15875	VFG002351(gb| WP_005160463)	64.964	(flgB) flagellar basal-body rod protein FlgB [Flagella (VF0394) - Motility (VFC0204)] [Yersinia enterocolitica subsp. enterocolitica 8081]
LIMFOD_00955	VFG041016(gb| NP_250349)	64.634	(tssC) type VI secretion system contractile sheath large subunit [HSI-2 (VF0943) - Effector delivery system (VFC0086)] [Pseudomonas aeruginosa PAO1]
LIMFOD_00950	VFG049914(gb| WP_000037389)	64.458	(tssB) Type VI secretion system protein TssB [T6SS (VF0579) - Effector delivery system (VFC0086)] [Shigella sonnei Ss046]
LIMFOD_04455	VFG000077(gb| NP_465991)	64.433	(clpP) ATP-dependent Clp protease proteolytic subunit [ClpP (VF0074) - Stress survival (VFC0282)] [Listeria monocytogenes EGD-e]
LIMFOD_06870	VFG013368(gb| WP_005693225)	63.988	(rffG) dTDP-glucose 46-dehydratase [LOS (VF0044) - Immune modulation (VFC0258)] [Haemophilus influenzae Rd KW20]
LIMFOD_00950	VFG038378(gb| WP_011705712)	63.871	(vipA) Type VI secretion system contractile sheath small subunit TssB/VipA [T6SS (VF0480) - Effector delivery system (VFC0086)] [Aeromonas hydrophila subsp. hydrophila ATCC 7966]
LIMFOD_15950	VFG043212(gb| WP_005168761)	63.851	(motB) flagellar motor protein MotB [Flagella (VF0394) - Motility (VFC0204)] [Yersinia enterocolitica subsp. enterocolitica 8081]
LIMFOD_15870	VFG002509(gb| WP_004197251)	63.768	(flgC) flagellar basal body rod protein FlgC [Flagella (VF0430) - Motility (VFC0204)] [Burkholderia pseudomallei K96243]
LIMFOD_11295	VFG002454(gb| WP_004187417)	63.750	(bsaX) Type III secretion system protein BsaX [Bsa T3SS (VF0428) - Effector delivery system (VFC0086)] [Burkholderia pseudomallei K96243]
LIMFOD_15900	VFG002522(gb| WP_004198639)	63.703	(flhA) flagellar biosynthesis protein FlhA [Flagella (VF0430) - Motility (VFC0204)] [Burkholderia pseudomallei K96243]
LIMFOD_09920	VFG013390(gb| WP_005651701)	63.670	(lpxA) UDP-N-acetylglucosamine acyltransferase [LOS (VF0044) - Immune modulation (VFC0258)] [Haemophilus influenzae Rd KW20]
LIMFOD_10500	VFG000104(gb| WP_000672505)	63.636	(acfB) accessory colonization factor AcfB [ACF (VF0127) - Others (VFC0346)] [Vibrio cholerae O1 biovar El Tor str. N16961]
LIMFOD_09930	VFG013374(gb| WP_005693262)	63.501	(lpxD) UDP-3-O-(3-hydroxymyristoyl) glucosamine N-acyltransferase [LOS (VF0044) - Immune modulation (VFC0258)] [Haemophilus influenzae Rd KW20]
LIMFOD_09860	VFG045346(gb| WP_011149697)	63.469	(IlpA) immunogenic lipoprotein A [IlpA (VF0513) - Adherence (VFC0001)] [Vibrio vulnificus YJ016]
LIMFOD_15905	VFG002357(gb| WP_005164474)	63.421	(flhB) flagellar biosynthetic protein FlhB [Flagella (VF0394) - Motility (VFC0204)] [Yersinia enterocolitica subsp. enterocolitica 8081]
LIMFOD_15810	VFG002495(gb| WP_004199882)	63.291	(fliQ) flagellar biosynthesis protein FliQ [Flagella (VF0430) - Motility (VFC0204)] [Burkholderia pseudomallei K96243]

LIMFOD_00325	VFG011430(gb| WP_002963616)	63.158	(acpXL) acyl carrier protein [LPS (VF0367) - Immune modulation (VFC0258)] [Brucella melitensis bv. 1 str. 16M]
LIMFOD_15935	VFG043378(gb| WP_000467802)	63.077	(cheV3) chemotaxis coupling protein CheV3 [Flagella (VF0051) - Motility (VFC0204)] [Helicobacter pylori 26695]
LIMFOD_15770	VFG038639(gb| WP_011705275)	62.911	(fliI) flagellum-specific ATP synthase [Polar flagella (VF0473) - Motility (VFC0204)] [Aeromonas hydrophila ML09-119]
LIMFOD_09915	VFG013384(gb| WP_010869121)	62.865	(lpxB) lipid-A-disaccharide synthase [LOS (VF0044) - Immune modulation (VFC0258)] [Haemophilus influenzae Rd KW20]
LIMFOD_00995	VFG038395(gb| WP_011705721)	62.804	(clpB) type VI secretion system ATPase ClpV1 [T6SS (VF0480) - Effector delivery system (VFC0086)] [Aeromonas hydrophila subsp. hydrophila ATCC 7966]
LIMFOD_15915	VFG038699(gb| WP_010634617)	62.698	(cheY) chemotaxis protein CheY [Polar flagella (VF0473) - Motility (VFC0204)] [Aeromonas hydrophila ML09-119]
LIMFOD_15795	VFG002018(gb| WP_010947517)	62.651	(fliN) flagellar motor switch protein FliN [Flagella (VF0157) - Motility (VFC0204)] [Legionella pneumophila subsp. pneumophila str. Philadelphia 1]
LIMFOD_15770	VFG001254(gb| NP_249795)	62.588	(fliI) flagellum-specific ATP synthase FliI [Flagella (VF0273) - Motility (VFC0204)] [Pseudomonas aeruginosa PAO1]
LIMFOD_15855	VFG002347(gb| WP_011816608)	62.550	(flgF) flagellar basal-body rod protein FlgF [Flagella (VF0394) - Motility (VFC0204)] [Yersinia enterocolitica subsp. enterocolitica 8081]
LIMFOD_15970	VFG000575(gb| NP_462663)	62.445	(mgtC) Salmonella virulence protein MgtC [MgtC (VF1365) - Nutritional/Metabolic factor (VFC0272)] [Salmonella enterica subsp. enterica serovar Typhimurium str. LT2]
LIMFOD_15925	VFG043208(gb| WP_005164483)	62.191	(cheR) chemotaxis methyltransferase CheR [Flagella (VF0394) - Motility (VFC0204)] [Yersinia enterocolitica subsp. enterocolitica 8081]
LIMFOD_09620	VFG000885(gb| WP_000265729)	62.128	(papD) chaperone protein PapD [P fimbriae (VF0220) - Adherence (VFC0001)] [Escherichia coli CFT073]
LIMFOD_15775	VFG002332(gb| WP_005160389)	62.069	(fliJ) flagellar protein FliJ [Flagella (VF0394) - Motility (VFC0204)] [Yersinia enterocolitica subsp. enterocolitica 8081]
LIMFOD_05165	VFG000270(gb| WP_000724295)	61.863	(ureB) urease beta subunit UreB, urea amidohydrolase [Urease (VF0050) - Stress survival (VFC0282)] [Helicobacter pylori 26695]
LIMFOD_10980	VFG012095(gb| WP_003435012)	61.860	(groEL) chaperonin GroEL [GroEL (VF0594) - Adherence (VFC0001)] [Clostridium difficile 630]
LIMFOD_08335	VFG013346(gb| WP_005693178)	61.856	(galU) glucosephosphate uridylyltransferase [LOS (VF0044) - Immune modulation (VFC0258)] [Haemophilus influenzae Rd KW20]
LIMFOD_15840	VFG002515(gb| WP_004550967)	61.625	(flgI) flagellar P-ring protein precursor FlgI [Flagella (VF0430) - Motility (VFC0204)] [Burkholderia pseudomallei K96243]
LIMFOD_15770	VFG043340(gb| WP_011946712)	61.412	(fliI) flagellum-specific ATP synthase FliI [Flagella (VF0157) - Motility (VFC0204)] [Legionella pneumophila subsp. pneumophila str. Philadelphia 1]
LIMFOD_11680	VFG001381(gb| YP_177728)	61.268	(icl) Isocitrate lyase Icl (isocitrase) (isocitratase) [Isocitrate lyase (VF0253) - Others (VFC0346)] [Mycobacterium tuberculosis H37Rv]
LIMFOD_00945	VFG041013(gb| NP_250203)	61.176	(hcpA) Hcp family type VI secretion system effector [HSI-2 (VF0943) - Effector delivery system (VFC0086)] [Pseudomonas aeruginosa PAO1]
LIMFOD_18260	VFG041013(gb| NP_250203)	61.176	(hcpA) Hcp family type VI secretion system effector [HSI-2 (VF0943) - Effector delivery system (VFC0086)] [Pseudomonas aeruginosa PAO1]
LIMFOD_04360	VFG049133(gb| WP_004177236)	61.069	(acrA) acriflavine resistance protein A [AcrAB (VF0568) - Antimicrobial activity/Competitive advantage (VFC0325)] [Klebsiella pneumoniae subsp. pneumoniae NTUH-K2044]
LIMFOD_19875	VFG013286(gb| WP_005694325)	61.012	(galE) UDP-glucose 4-epimerase [LOS (VF0044) - Immune modulation (VFC0258)] [Haemophilus influenzae Rd KW20]
LIMFOD_05180	VFG000269(gb| WP_000779223)	61.000	(ureA) urease alpha subunit UreA [Urease (VF0050) - Stress survival (VFC0282)] [Helicobacter pylori 26695]
LIMFOD_01085	VFG013236(gb| WP_005693864)	60.956	(kdsB) 3-deoxy-manno-octulosonate cytidylyltransferase [LOS (VF0044) - Immune modulation (VFC0258)] [Haemophilus influenzae Rd KW20]
LIMFOD_15815	VFG002340(gb| WP_005168816)	60.769	(fliR) flagellar biosynthetic protein FliR [Flagella (VF0394) - Motility (VFC0204)] [Yersinia enterocolitica subsp. enterocolitica 8081]
LIMFOD_15915	VFG043012(gb| NP_250147)	60.656	(cheY) chemotaxis protein CheY [Flagella (VF0273) - Motility (VFC0204)] [Pseudomonas aeruginosa PAO1]
LIMFOD_17915	VFG002228(gb| WP_002963677)	60.606	(wzt) O-antigen export system ATP-binding protein [LPS (VF0367) - Immune modulation (VFC0258)] [Brucella melitensis bv. 1 str. 16M]
LIMFOD_12850	VFG041013(gb| NP_250203)	60.588	(hcpA) Hcp family type VI secretion system effector [HSI-2 (VF0943) - Effector delivery system (VFC0086)] [Pseudomonas aeruginosa PAO1]
LIMFOD_07920	VFG013400(gb| WP_005693429)	60.571	(rfaF) ADP-heptose-LPS heptosyltransferase II [LOS (VF0044) - Immune modulation (VFC0258)] [Haemophilus influenzae Rd KW20]
LIMFOD_14490	VFG012627(gb| WP_000910392)	60.526	(chuU) heme permease protein ChuU [Chu (VF0234) - Nutritional/Metabolic factor (VFC0272)] [Escherichia coli O157:H7 str. EDL933]
LIMFOD_14490	VFG000922(gb| WP_000910402)	60.526	(chuU) heme permease protein ChuU [Chu (VF0227) - Nutritional/Metabolic factor (VFC0272)] [Escherichia coli CFT073]
LIMFOD_18240	VFG039536(gb| NP_820549)	60.494	(CBU_1566) Coxiella Dot/Icm type IVB secretion system translocated effector [T4SS secreted effectors (VF0696) - Effector delivery system (VFC0086)] [Coxiella burnetii RSA 493]
LIMFOD_15805	VFG001259(gb| NP_250137)	60.476	(fliP) flagellar biosynthetic protein FliP [Flagella (VF0273) - Motility (VFC0204)] [Pseudomonas aeruginosa PAO1]

LIMFOD_13980	VFG000463(gb|NP_460019)	60.465	(sodCI) Gifsy-2 prophage: superoxide dismutase precursor (Cu-Zn) [SodCI (VF0109) - Stress survival (VFC0282)] [Salmonella enterica subsp. enterica serovar Typhimurium str. LT2]
LIMFOD_15935	VFG002529(gb|WP_011205290)	60.390	(tsr) methyl-accepting chemotaxis protein I [Flagella (VF0430) - Motility (VFC0204)] [Burkholderia pseudomallei K96243]
LIMFOD_15795	VFG002492(gb|WP_004184995)	60.317	(fliN) flagellar motor switch protein FliN [Flagella (VF0430) - Motility (VFC0204)] [Burkholderia pseudomallei K96243]
LIMFOD_14490	VFG013069(gb|WP_005019013)	60.197	(shuU) permease of iron compound ABC transport system [Shu (VF0256) - Nutritional/Metabolic factor (VFC0272)] [Shigella dysenteriae Sd197]
LIMFOD_11300	VFG003519(gb|WP_032903070)	60.190	(ysaR) EscR/YscR/HrcR family type III secretion system export apparatus protein [Ysa TTSS (VF1025) - Effector delivery system (VFC0086)] [Yersinia enterocolitica subsp. enterocolitica 8081]

Table S10. BURST analysis of virulence type vST138 *P. miralis* (n=162) in the PubMLST database.

BURST analysis				
**ST**	**No. of isolates**	**SLV**	**DLV**	**SAT**
93*	22	6	4	
135	118	5	3	2
140	4	4	5	1
207	8	1	4	5
263	1	3	5	2
326	1	1	5	4
439	1	3	5	2
461	1	1	5	4
516	1	1	3	6
540	2	1	6	3
575	2	2	7	1
